# Alleviation of Multidrug Resistance by Flavonoid and Non-Flavonoid Compounds in Breast, Lung, Colorectal and Prostate Cancer

**DOI:** 10.3390/ijms21020401

**Published:** 2020-01-08

**Authors:** Teodora Costea, Oana Cezara Vlad, Luminita-Claudia Miclea, Constanta Ganea, János Szöllősi, Maria-Magdalena Mocanu

**Affiliations:** 1 Department of Pharmacognosy, Phytochemistry and Phytotherapy, “Carol Davila” University of Medicine and Pharmacy, 020021 Bucharest, Romania; teodora.costea@umfcd.ro; 2 Department of Biophysics, “Carol Davila” University of Medicine and Pharmacy, 020021 Bucharest, Romania; vlad.oana18@gmail.com (O.C.V.); constanta.ganea@gmail.com (C.G.); 3 Department of Biophysics and Cellular Biotechnology, “Carol Davila” University of Medicine and Pharmacy, 020021 Bucharest, Romania; luminita.miclea@umfcd.ro; 4 Research Excellence Center in Biophysics and Cellular Biotechnology, “Carol Davila” University of Medicine and Pharmacy, 020021 Bucharest, Romania; 5 Department of Biophysics and Cell Biology, Faculty of Medicine, University of Debrecen, 4032 Debrecen, Hungary; szollo@med.unideb.hu; 6 MTA-DE Cell Biology and Signaling Research Group, Faculty of Medicine, University of Debrecen, 4032 Debrecen, Hungary

**Keywords:** chemoresistance, malignancy, phenolic compounds

## Abstract

The aim of the manuscript is to discuss the influence of plant polyphenols in overcoming multidrug resistance in four types of solid cancers (breast, colorectal, lung and prostate cancer). Effective treatment requires the use of multiple toxic chemotherapeutic drugs with different properties and targets. However, a major cause of cancer treatment failure and metastasis is the development of multidrug resistance. Potential mechanisms of multidrug resistance include increase of drug efflux, drug inactivation, detoxification mechanisms, modification of drug target, inhibition of cell death, involvement of cancer stem cells, dysregulation of miRNAs activity, epigenetic variations, imbalance of DNA damage/repair processes, tumor heterogeneity, tumor microenvironment, epithelial to mesenchymal transition and modulation of reactive oxygen species. Taking into consideration that synthetic multidrug resistance agents have failed to demonstrate significant survival benefits in patients with different types of cancer, recent research have focused on beneficial effects of natural compounds. Several phenolic compounds (flavones, phenolcarboxylic acids, ellagitannins, stilbens, lignans, curcumin, etc.) act as chemopreventive agents due to their antioxidant capacity, inhibition of proliferation, survival, angiogenesis, and metastasis, modulation of immune and inflammatory responses or inactivation of pro-carcinogens. Moreover, preclinical and clinical studies revealed that these compounds prevent multidrug resistance in cancer by modulating different pathways. Additional research is needed regarding the role of phenolic compounds in the prevention of multidrug resistance in different types of cancer.

## 1. Introduction

Cancer is one of the leading cause of death worldwide. It is usually caused by genome instability and mutations, which may be inherited, induced by environmental factors or represent a consequence of DNA replication errors [1]. The signature characteristics of cancer are represented by: a high rate cellular multiplication escaping growth inhibitors, cell migration inducing subsequent metastasis, stimulation of local new blood vessel formation (angiogenesis), the capacity to resist cell senescence and death signals leading to inflammation, and an almost unlimited self-replicating capacity [2]. 

The number of cancer cases is expected to increase rapidly as populations grow, age and adopt negative lifestyle behaviors (smoking, lack of physical activity, Western diet) that increase cancer risk [3,4]. Lung, breast, colorectal and prostate cancer are considered to be the most prevalent types of cancer among population [3]. 

For women, *breast cancer* is the most common diagnosed malignancy, followed by cervix or uterine cancer [3]. In Europe, it is estimated that breast cancer affects more than one in 10 women and accounts for more than 28% of female cancers [5]. Risk factors for breast cancer include unmodifiable factors and lifestyle factors. Among unmodifiable factors, age (above 40 years), family history of cancer in first-degree relatives, hormonal profile (late menopause, early menarche), dense breast tissue, race and genetics (mutation in breast cancer susceptibility genes—*BCRA1* and *BCRA2* genes, *TP53*, genetic polymorphisms in genes encoding enzymes involved in estrogen metabolism pathways COMT, CYP1A1, CYP1B1, estrogen receptors ERα/ERβ, CYP17A1 and CYP19A1) are of great importance. Lifestyle factors include nulliparity, use of birth control pills, induced abortion or obesity [6,7,8,9,10]. Although breast cancer usually appears in pre- and post-menopausal women, recently new cases have occurred even in young women, below 35 years. This represents a serious concern, due to higher incidence of advanced stages at diagnosis and poorer five-year survival rate [11] compared to older women. Breast cancer represents a heterogeneous disease and it is clinically divided into three basic subtypes: (I) based on the level of expression of estrogen and progesterone receptors, (II) based on the human epidermal growth factor 2 (HER2) and (III) a third subtype, when neither estrogen, progesterone or HER2 is expressed (triple negative breast cancer [12]. Breast tumors expressing hormone receptors (mainly estrogen) are classified as luminal breast type (luminal A and B). Luminal A subtype has a better prognosis compared to luminal-B type, which is more aggressive, has a higher recurrence and an increased expression of growth receptor signaling molecules, such as epidermal growth factor (EGF), fibroblast growth factor (FGF), nerve growth factor (NGF), hepatocyte growth factor receptor (HGFR/MET) and Wnt/β-catenin [13]. Increased growth receptor signaling genes is also observed for triple breast negative cancer [14]. Nowadays, mammography represents the golden standard for breast cancer screening [15]. 

*Lung cancer* is the most common cancer in men worldwide, and the fourth most frequent cancer in women [16]. Lung cancer is often divided into four major types due to distinct clinic-pathological features: small cell lung cancer (SCLC) and non-small cell lung cancer (NSCLC), which is further divided into squamous cell carcinoma (SCC), adenocarcinoma and large cell carcinoma [17]. Risk factors for lung cancer include smoking, environmental exposure to tobacco, radon, cooking oil vapors or hormonal factors (mainly in women). Moreover, genetic factors play a major role in lung cancer etiology [18,19,20]. 

*Colorectal cancer* is one of the most preventable and treatable cancers if detected early; however, it has a multifactorial etiology. The hallmark of colorectal cancer is the presence of serrated or adenomatous polyps (adenoma) that usually occur in proximal or distal colon [21]. Besides adenomas, patients with colorectal cancer have multiple aberrant crypt foci, which are microscopic mucosal abnormalities involved in early carcinogenesis [22]. Main risk factors include alterations of gut microbiota [23], Western diet [24], obesity, hormonal status or chronic inflammatory bowel diseases [25]. Genetic factors such as mutations in *KRAS*, *BRAF*, *PI3K* genes and polymorphisms in nucleic acid-binding protein 1, laminin γ 1, cyclin D2, T-box 3 are also involved in colorectal cancer etiology [26,27].

*Prostate cancer* is the second most prevalent type of cancer among men, besides lung cancer. The majority of prostate cancers originate from luminal cells and do not have a neuroendocrine origin [28]. Risk factors for prostate cancer include age, obesity, other diseases (diabetes), lifestyle behaviors (diet, lack of physical activity) and sexually transmitted diseases [29]. Main characteristics of prostate cancer include activation of androgen receptor signaling, elevated lymphocyte infiltration and activation of inflammatory pathways [30].

The above-mentioned cancer types have a common feature, which is represented by multidrug resistance (MDR) to chemotherapeutic treatments [13,28,31]. Due to toxicity and lack of specificity of synthetic MDR agents, recent researches have focused on beneficial effects of natural compounds in overcoming MDR in cancer. According to recent research, polyphenols might overcome MDR through various mechanisms, which will be further discussed in our work [32,33,34,35].

Polyphenols are considered as important dietary components with biological activity due to a wide range of health benefits: antioxidant, anti-inflammatory, anti-carcinogenic, immunomodulatory, etc. [36,37]. Epidemiological studies have shown that intake of food rich in phenolic compounds have chemopreventive effects for cardiovascular, neurodegenerative diseases, cancer, obesity or diabetes [38]. Cancer chemopreventive effects of polyphenols are the consequence of antioxidant capacity, inhibition of proliferation, survival, angiogenesis and metastasis, modulation of immune and inflammatory responses or inactivation of pro-carcinogens [39]. 

Polyphenols comprise a variety of compounds with a wide range of chemical structures, ranging from single molecules to high molecular weight polymers. Polyphenols have at least one aromatic ring and are classified as flavonoids and non-flavonoids in correlation with the number of aromatic ring [38,40]. Flavonoids share a C_6_-C_3_-C_6_ structural backbone and are further classified into flavones, flavonols, flavanones and flavan-3-ols [38]. Isoflavones, are also members of flavonoids family [38]. Non-flavonoid compounds include phenolcarboxylic acids (hydroxy-benzoic/hydroxy-cinnamic acids), ellagitannins, lignans, stilbenes and other phenolic compounds (curcumin, gingerol) [40]. A selective list of polyphenols, which are frequently studied for overcoming MDR in breast, lung, prostate and colorectal cancer, is presented in Table 1.

## 2. Mechanism of Multidrug Resistance in Cancer

**Figure 1:**
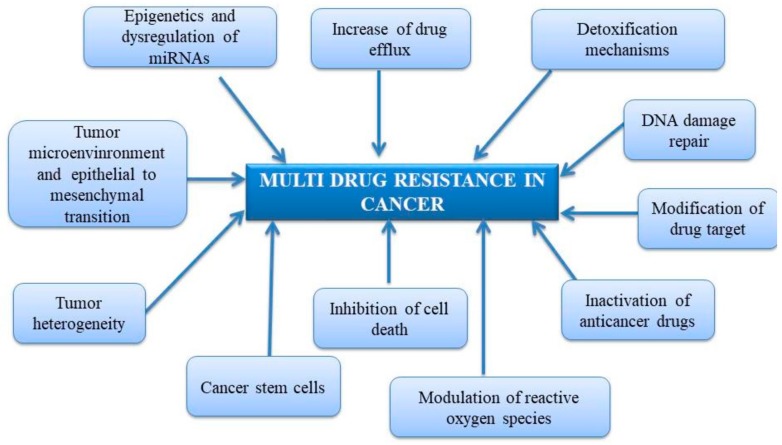
Mechanisms of multidrug resistance in cancer.

Earlier papers reported only few mechanisms responsible for MDR in cancer (Figure 1), such as (i) increased drug efflux through membrane pumps, (ii) detoxification mechanisms based on glutathione transferases activity, (iii) DNA damage repair that initially may be considered as an ally and further can turn into a resistant tool, and (iv) drug inactivation [52]. However, recent papers described extended lists of mechanisms responsible for drug resistance in malignancy (Figure 1) such as modification of drug target, inhibition of cell death, involvement of cancer stem cells, tumor heterogeneity, tumor microenvironment, epithelial to mesenchymal transition, epigenetic variations, dysregulation of miRNAs and modulation of reactive oxygen species [53,54,55]. 

### 2.1. Increase of Drug Efflux

At the plasma membrane level, the large family of ATP-binding cassette (ABC) transporter proteins is responsible mainly for the drug efflux [56]. ABC transporters consist of two transmembrane domains and two intracellular nucleotide-binding domains. It is the nucleotide binding domains that bind ATP and hydrolyze it to ADP providing the plasma membrane pump with energy required to export xenobiotic compounds [57]. There are 49 known *ABC* genes organized in subfamilies, from A to G, respectively 12 *ABCA*, 11 *ABCB*, 13 *ABCC*, 4 *ABCD*, 1 *ABCE*, 3 *ABCF* and 5 *ABCG* [58]. The most studied ABC transporters are multidrug-resistance protein 1 (MDR1)/permeability-glycoprotein (P-pg)/ABCB1, MDR-associated protein 1 (MRP1) and breast cancer resistance protein (BCRP)/ABCG2 [56,59]. The majority of ABC transporters are localized in the liver, kidney, intestine, but they can have ubiquitous localization as well [56,59,60]. 

High levels of MDR1 are expressed in colorectal cancer [61], hepatocarcinoma [62], breast cancer [63], lung cancer [64] or prostate cancer [65]. Overexpression of ABC transporters in cancer is mediated by (i) increased activity of proteins involved in the MAPK (HRas, ERK1/2, JNK), PI3K/AKT, mTOR, JNK, PKC signaling pathways, (ii) activation of EGF/FGF growth factors [54,66,67,68], (iii) nuclear localization of Y-box binding protein 1 (YB-1) in solid tumors [69,70], (iv) increased COX-2 activity [71], (v) activation of VEGF2 (vascular endothelial growth factor receptor 2) by VEGF in tumor microenvironment [70], (vi) activation of nuclear receptors PXR and CAR [72,73,74] and (vii) hypoxia [75]. According to recent studies inhibition of ERK1/2, NF-κB pathways and increased sensitivity to *all*-trans retinoic acid (a ligand of retinoic acid receptors RARs) render cancer cells more sensitive to chemotherapeutic agents, due to reduced P-gp mediated efflux activity [54,76,77]. 

Moreover, extensive studies have shown a strong correlation between ABC transporters activity and *TP53* tumor suppressor gene [78,79]. It is well known that *TP53* mutations occur in almost 50% of cancers and are involved in inhibition of apoptosis [80]. According to Sullivan G. and his co-workers *TP53* mutations become increasingly frequent as prostate cancer advances in stage and this is strongly correlated with increased MRP1 expression [79]. 

Several chemotherapeutic agents (doxorubicin, daunorubicin, vincristine, vinblastine, actinomycin D, paclitaxel, docetaxel, etoposide) and molecular targeted anticancer compounds (i.e., tyrosine kinase inhibitors, such as imatinib, erlotinib, sunitinib) are substrates for MDR1 [81,82,83,84] and this fact has negative impact on drug efflux in malignant cells. In this context, many attempts have been reported to overcome MDR. 

Two main strategies have been employed to prevent drug resistance mediated by ABC protein transporters, namely (i) co-administration of MDR1 inhibitors with chemotherapeutical drugs with the aim to increase intracellular accumulation of drug and (ii) substrate competition by co-administration of MDR1 substrate together with the anticancer drug [85]. Some of the first modulators of MDR1 identified are calcium influx blockers (i.e., verapamil, nicardipine nifedipine), which increased the cytotoxicity of anticancer drugs in cancer cell lines [86,87,88]. Regrettably, the results from preclinical studies were difficult to apply in clinical trials for several reasons (i) necessity of higher concentrations, which in turn induced systemic toxicity, (ii) low selectivity and specificity due to the expression of the target in different tissues or (iii) low efficiency due to functional redundancy of ABC protein transporter family [85]. Recently, PPAR δ ligands (rosiglitazone and pioglitazone) were found to inhibit drug resistance in breast cancer cells by internalization of ABCG2 to cytoplasm [89]. Further research studies are needed to understand the molecular mechanism and to identify the optimal doses of MDR1 inhibitors for the development of new inhibitors of ABC protein transporters. 

### 2.2. Detoxification Mechanisms and Inactivation of Anticancer Drugs

Downregulation or mutations in the proteins or enzymes involved in activation of chemotherapeutic agents can be responsible for drug resistance [90]. For example, in tumor cells resistant to capecitabine, the gene responsible for the synthesis of thymidine phosphorylase, an enzyme responsible for generation of the nucleotides, can be inactivated by hypermethylation [91]. Carbonyl reduction of doxorubicin induced by aldo-keto reductase is responsible for transformation of doxorubicin into doxorubicinol, which is an inactive form. Administration of both chemotherapeutic drugs and inhibitors of aldo-keto reductase is recommended to overcome inactivation of doxorubicin and to increase its therapeutic activity [92]. 

Other important pathways of drug inactivation involve the CYP450 system (mainly CYP2B6, CYP2C9, CYP2C19, CYP2D6), glutathione-S-transferase (GST) superfamily or uridine diphospho-glucuronosyltransferase (UGT) superfamily [54]. For example, CYP2D6 polymorphism is involved in tamoxifen variability among patients with breast cancer, since CYP2D6 is involved in tamoxifen metabolization to 4-hydroxytamoxifen and endoxifen, both of which display higher anti-estrogenic activity [93]. Some of the first reports, reconfirmed later on, indicated that resistance to platinum could occur through drug inactivation by thiol glutathione, which activates the detoxification system (GST) [94,95]. It was reported that resistance to other chemotherapeutic agents (doxorubicin, tamoxifen, epirubicin), commonly used to treat breast cancer, is mediated by the polymorphisms in UGT superfamily [96].

### 2.3. DNA Damage Repair

Several chemotherapeutic drugs interfere with DNA synthesis with the aim to induce senescence, apoptosis or cell cycle arrest in cancer cells [97]. DNA-damaging compounds with anticancer properties can act through different mechanisms such as inducing DNA crosslinking (i.e., cisplatin, carboplatin, oxaliplatin), preventing DNA synthesis (i.e., antimetabolites that inhibit the activity of dihydropholate reductase) or inhibiting topoisomerase activity (i.e., doxorubicin, daunorubicin) [98]. Nevertheless, these compounds do not have a specific tumor target and the selectivity of anticancer drugs is based on the rate of cell cycling. Tumor cells have a rapid cycling compared to normal cells and DNA damage response proteins (DDR) do not have enough time to repair DNA lesions [99]. The major mechanisms of DNA repair pathways in response to chemotherapy are elegantly and thoroughly explained elsewhere [99]. Briefly, these processes include (i) mismatch repair (MMR) mechanisms which remove mis-incorporated nucleotides during DNA replication [100]; (ii) nucleotide excision repair (NER) which removes bulky DNA lesions, such as DNA adducts [101]; (iii) base excision repair (BER) that corrects small base lesions which occur after DNA damage produced by oxidation, deamination or alkylation [102]; (iv) homologous recombination (HR) which repairs DNA double-stranded breaks and inter-strand crosslinks [103]; (v) non-homologous end-joining (NHEJ) with the aim to repair double-stranded breaks [104]. 

Recent reports demonstrate that MDR to platinum drugs in cancer cell lines, implicates multiple DDR pathways including HR, transcription-coupled NER and BER [105]. MutL homolog 1 (MLH1) and MutL homolog 2 (MLH2)—proteins belonging to MMR system—have been evaluated by immunohistochemistry from patients with colorectal cancer and 10% of these patients presented MMR deficiency. Administration of 5-fluorouracil induced the improvement of survival only in patients without MMR deficiency, demonstrating the association between dysregulation in MMR processes and multidrug resistance [106]. Due to constantly improving technology, the researchers might carry out genomic screening with the aim to identify potential DNA therapeutic targets responsible for MDR in malignancies. 

### 2.4. Modification of Drug Target

A drug’s efficacy strongly depends on its molecular target. Alteration of these targets by means of different mechanisms (i.e., mutations) may lead to drug resistance [54]. One of the most studied mechanisms of drug resistance in respect with modification of the drug target is focused on epidermal growth factor receptor (EGFR) [107]. In non-small-cell lung cancer (NSCLC) activation mutations of EGFR in the tyrosine kinase domain had been identified. Small molecule inhibitors such as gefitinib and erlotinib are known to neutralize these modifications [107]. Nevertheless, after two years of gefitinib treatment the disease can relapse, due to occurrence of secondary mutation (T790M) in EGFR [108]. Second generation of EGFR tyrosine inhibitors (i.e., ponatinib) had been created to act against EGFR(T790M), but increased toxicity caused withdrawal of the drug from the market [109]. Due to ability of cancer cells to survive by occurrence of additional mutations, new generations of tyrosine kinase inhibitors (TKI) against EGFR or other molecular targets are needed to be developed to overcome MDR and side effects associated with anticancer therapy. 

### 2.5. Inhibition of Cell Death

Cancer cells escape cell death using several mechanisms such as dysregulation of apoptosis, inhibition of other non-apoptotic processes (i.e., autophagy, etc.) or stimulation of alternative survival pathways [53]. The most studied mechanisms, which allow cancer cells to evade cell death and to acquire MDR, are the disturbance of apoptosis and inhibition of autophagy. The main proteins involved in apoptosis are the caspases, which can be activated by both intrinsic (in the mitochondria) and extrinsic (through tumor necrosis family factors that bind to cell death receptors) pathways [93,110,111,112]. 

Mechanisms of drug resistance due to apoptosis deregulation include: (i) imbalance of Bcl-2 family members (downregulation of pro-apoptotic proteins Bax and upregulation of anti-apoptotic proteins BCL-X_L_, BCL-2), (ii) altered apoptotic regulators (downregulation of caspase−3, −8, −9 and upregulation of inhibitors of apoptosis proteins such as XIAP, FLIP, survivin), (iii) upregulation of ubiquitin binding proteins (sharpin), which regulates Bcl-2 and survivin [113], (iv) decreased activity of p53 and PTEN [80,90,93], (v) decreased activity of cytochrome C and Smac/DIABLO (which are responsible for caspases activation) [114,115], (vi) deregulated activity of cyclin-dependent kinases (CDK), protein tyrosine kinases (Her2,/neu, Her3, Her4) [116] or different signaling pathways (GSK-3; STAT3, PI3K/AKT, mTOR) [115,117,118] or (vii) amplification of gene expression of *CYCLINS* (*A1, D1*) [119]. Checkpoint kinases (Chk1, Chk2), which are modulated by serine/threonine protein kinases (ATR), also play a major role in apoptosis since they promote activation of p21 and p53, which induce cell cycle arrest [120].

Autophagy is involved in MDR through increased activity of AMP-protein kinase (AMPK), beclin-1 and activation of autophagy lysosomes systems (ALP) [75,93]. ALP in most tumors may enhance the MDR phenotype through a protein clearance mechanism [75]. Elevated autophagy lysosomes systems are involved in EGFR inhibitors (gefitinib, erlotinib), mTOR inhibitors (temsirolimus) or targeted therapy (imatinib) chemoresistance [75].

It is reasonable to assume that genes, mRNA and proteins involved in disturbed apoptotic and autophagy processes are considered optimal targets to overcome multidrug resistance in malignant tumors. Against anti-apoptotic BCL-2 proteins both antisense oligonucleotides (i.e., oblimersen sodium) that target BCL-2 mRNA and small molecules which can interact with BH3 domains have been developed [121,122]. The last category might be divided in small molecules with BH3 mimetic activity (i.e., ABT-737, navitoclax/ABT-263/oral version of ABT-737) and small molecules with BH3 putative mimetic action (i.e., gossypol, obatoclax/a pan-BCL-2 inhibitor, etc.) [123]. 

Nevertheless, several mechanisms of drug resistance developed by cancer cells hindered the successful application of anti-apoptotic drugs in patients. For instance, clinical studies on combinatorial administration of several chemotherapeutics (i.e., dacarbazine, fludarabine, cyclophosphamide) and oblimersen did not bring favorable results in patients [122,124]. Polymorphism of BCL-2-like protein 11 (BIM) with different splicing variants resulted in lack of BH3 domain and resistance to targeted therapy in NSCLC positive for EGFR [125]. 

Stimulation of pro-apoptotic death receptors (i.e., DR4, DR5) localized in plasma membrane demonstrated in vitro and in vivo anti-proliferative activity, but clinical results have been unsatisfactory [126,127]. Nevertheless, preclinical experiments with the aim to test synergism of combinatorial administration of death receptors agonists and other anti-cancer drugs are under evaluation [128,129]. Recently, inhibitors of CDK (roscovitine, terameprocol, flavopiridol) are under investigation in different MDR cancers [116]. 

Moreover, it was shown that PPAR δ agonists (rosiglitazone) sensitizes colorectal cancer cells to 5-FU by downregulation of Bcl-2 proteins and upregulation of Bax [130]. Inhibition of ALP using chloroquine and hydroxychloroquine is also under investigation in both preclinical and clinical studies [75]. 

Further preclinical experiments and successful clinical trials are needed to better understand the molecular mechanisms of anti-apoptotic/autophagy processes and to circumvent the drug resistance in cancer cells.

### 2.6. Cancer Stem Cells

There is increasing evidence that cancer stem cells (CSCs), a subpopulation of cells within the heterogenous tumor niche, are responsible for initiation of some primary tumors as well as metastasis and MDR [90,93]. CSCs are resistant to chemotherapy and radiotherapy given to their particular characteristics such as increased DNA damage repair, resistance to cell death mechanisms, evasion from immune response, adaptation to hypoxia and overexpression of MDR efflux pumps [93,131]. Several lines of action have been developed to overcome drug resistance in cancer stem cells. These include (i) new inhibitors against ABC transporters, (ii) antibodies conjugated with toxins or radioisotopes against ABC transporters, (iii) inhibitors of signaling pathways identified in cancer stem cells (i.e., Hedgehog signaling pathway) or (iv) activation of immune system against cancer stem cells [131,132]. In spite of the extensive efforts to address drug resistance in cancer stem cells there are still open questions needing to be answered. For instance, how is it possible that ABC transporters or Hedgehog signaling pathways can be targeted only in cancer stem cells and not in normal stem cells? In addition, recent papers underline the contribution of cancer niche as a crucial factor in drug resistance of CSCs [133,134]. Cancer associated fibroblasts stimulated 5-fluorouracil resistance in colon CSC by activating Wnt signaling [135] or autocrine generation of inflammatory factors, such as interleukin-6 induced trastuzumab resistance in HER2 positive breast CSC [136]. Besides addressing ABC transporters as therapeutic targets, CSC niche could represent a potential objective in further anticancer approaches with the aim to overcome MDR. 

### 2.7. Tumor Heterogeneity

Genetic instability allows survival of the best adaptable clonal populations of malignant cells, and this heterogeneity represents one of the reasons for the failure of anticancer therapy [137,138]. It is already recognized that tumor heterogeneity implies two distinct types of processes, (i) tumor inter-heterogeneity, with tumors affecting the same organ, but with different characteristics in each patient, and (ii) tumor intra-heterogeneity, with two branches, spatial and temporal heterogeneity [139]. Spatial heterogeneity is present in the same patient and it is characterized by different genotypes and phenotypes of the malignant clones in the primary and metastatic sites, while temporal heterogeneity expresses the changes which are taking place in the same tumor over the time [139]. In cancer cells overexpressing hepatocyte growth factor receptor (HGFR/MET), heterogeneity occurred as a molecular mechanism of drug resistance after chemotherapy [140]. Thus, after two years of targeted therapy against MET, two additional changes have been identified, *KRAS* mutation and co-amplification of *HER2* and/or *EGFR* genes [140]. Chronical administration of the chemotherapeutic drugs demonstrated that in one or two years the diseases relapsed due to the ability of cancer cells to generate new clones and to find alternative pathways to survive and proliferate [108,141]. 

In vitro and in vivo experiments have been performed to identify the culprit molecules or alternative pathways that confer drug resistance [142,143]. Escape of human epidermal growth factor receptor type 2 (HER2) from the inhibition with tyrosine kinase inhibitor (TKI) through alternative HER3 activation has been demonstrated in mammary cancer cell lines [142]. Not only in case of chemotherapy, but also in case of hormone therapy the existence of adaptive mechanisms and acquired resistance has been reported [144,145]. Increased survival and reduction of prostate serum antigen (PSA) levels are described after androgen deprivation by enzalutamide in prostate cancers [146]. However, secondary mutations are identified in castration-resistant prostate cancers after administration of enzalutamide [147]. Similar to hormone therapy against prostate cancer, first results about administration of tamoxifen in estrogen receptor (ER) positive breast cancer patients have been promising and there are recommendations to increase the administration from five to 10 years [148]. Notably, chronical administration of hormone therapy can cause resistance and most frequently alternative signaling pathways activated in estrogen resistant breast cancer are plasma membrane tyrosine kinase receptors, such as EGFR, HER2, IGF-1R or downstream kinases, such as ERK1/2, PI3K/AKT [144,149].

Increased exposure of the malignant cells to different anticancer agents amplifies the heterogeneity of the tumor and several overcoming therapies against drug resistance are proposed [139]. These include (i) combination therapy against single target (i.e., TKI afatinib against EGFR and monoclonal antibody cetuximab against EGFR) [150] or against multiple targets (i.e., a third generation TKI of EGFR(T790M) and navitoclax an inhibitor of ABC transporters) [151]; (ii) sequential therapy to reduce the toxicity induced by combination of chemotherapeutic agents [152] or (iii) targeted therapy after identification of genetic markers (i.e., patients with EGFR(T790M) mutation which can benefit from osimertinib treatment compared to patients with activating mutations in EGFR who can benefit by gefinitib/erolotinib/afatinib administration) [153]. New experimental studies and different therapeutic approaches are required to find the optimal way to interfere with development of tumor malignancy. 

### 2.8. Tumor Microenvironment (TME)

In spite of the fact that TME is formed from non-malignant structures (i.e., cancer associated fibroblast, immune cells, adipocytes, extracellular matrix molecules, blood and lymphatic vessels, and mesenchymal cells), in most cases they are considered as tumor-promoting factors [154]. Main mechanisms involved in TME role in MDR are (i) abnormal tumor vasculature (promotion of angiogenesis and overexpression of VEGF), (ii) hypoxia, (iii) decreased pH (due to glycolysis), (iv) alterations in the expression of tumor suppressors and oncogenes [155,156,157,158] and (v) modulation of different signaling pathways (mTOR, ERK1/2) and growth-factors (FGF) [159]. Among TME factors, hypoxia plays a major role in lung, colorectal, breast and prostate cancers MDR [155,160,161,162,163]. Hypoxia induces HIF-1 (hypoxia-inducible factor 1) in tumor cells, upregulates the release of pro-angiogenic factors, increases the expression of growth-factor receptors (CXCR4) and MDR proteins (P-gp) [164]. Moreover, the relatively low pH values—a direct consequence of hypoxia—are responsible for reduced cellular uptake of chemotherapeutic agents [165].

Other important factors of TME which promote MDR are the overexpression of fatty acid synthase (FASN) and fatty acid-binding proteins (FBAP4, FBAP5, FBAP9) in breast/prostate tumor cells [166,167]. FSAN is required for de novo synthesis of fatty acids and is correlated with poor prognosis of cancer [166]. Overexpression of FASN may induce drug resistance by (i) altering the membrane composition, thus decreasing the influx of chemotherapeutic agents; (ii) upregulation of HER2 or (iii) inhibition of apoptosis [168,169]. 

According to recent research, the cellular components of the tumor stroma (fibroblasts, infiltrated immune cells or mesenchymal stromal cells) induce MDR through increased expression of cytokines (IL-6, IL-8, IL-18, IL-17), overexpression of HER2 and loss of PTEN (tumor suppressor gene) activity [54,170,171,172]. To date several small molecule inhibitors and antibodies against tumor stroma are in clinical trials (prinomastat, saridegib, bevacizumab, etc.) [171].

### 2.9. Epithelial to Mesenchymal Transition (EMT)

Tumor microenvironment plays a major role in cancer cells ability to develop further features such as cell transition from epithelial to mesenchymal phenotype. This transformation gives them the advantage to migrate to secondary sites [173]. EMT is considered to be an important mechanism by which tumors become metastatic and multidrug-resistant [54,174]. Drug resistance developed after administration of EGFR-target therapy (i.e., erlotinib and cetuximab) has been reported to be connected with EMT features [175].

The PI3K/AKT is one of the most important signaling pathways that mediates the process of EMT through (i) direct activation of transcription factors (twist 1, 2) which increases the expression of mesenchymal markers (N-cadherin), decreases the expression of epithelial markers (E-cadherin, claudin, occluding) and upregulates *AKT* gene, which is involved in drug resistance in breast cancer, (ii) increased activity of integrin-linked kinase (which downregulates E-cadherin) and (iii) activation of matrix-degrading proteases (MMP2, MMP9) [55,174]. Moreover, other factors are also involved in EMT activation such as growth factors (FGF, EGF, TGF-β), adhesion molecules (ICAM-1), signaling pathways (NF-κB, Wnt/β-catenin, Notch), overexpression of EMT transcription factors (slug, snail) and members of heat-shock proteins family (such as glucose regulated protein 78 (GRP78)) [53,172,174,176]. Notably, due to the correlation between drug resistance and acquisition of EMT phenotype (i.e., EMT modified cells appear similar to CSC as a result of their high levels of ABC transporters), targeting EMT might represent a new toll to circumvent drug resistance in cancer [177]. 

### 2.10. Epigenetic Variations

The main types of epigenetic mechanisms involved in cancer drug resistance are DNA methylation and histone alterations [54]. Aberrant DNA methylation is associated with genes encoding for proteins involved in cell differentiation, proliferation, apoptosis (MAPK, VEGF, Wnt/β-catenin, p15, p16, p53, APAF-1) or genes encoding drug transporters (MDR1) [90,93]. Moreover, epigenetic mechanisms can also affect the DNA repair system, since hypermethylation of hMLH1 gene is responsible for colorectal cancer [90]. 

Recently several studies revealed the important role of epigenetic regulator, polycomb repressive complex 2 catalytic component enhancer of zeste homolog 2 (EZH2), in neoplastic development and drug resistance in many types of cancer (gastric, lung, hepatic) [178]. According to Chang and co-workers, overexpression of EZH2 upregulates EMT transition and decreases sensitivity to several chemotherapeutic agents (i.e., cisplatin) [179]. Since epigenetic alterations might represent a viable anticancer and anti-drug resistance target, a large series of DNA methylation or histone deacetylases inhibitors have been generated. These comprise nucleoside analogs (i.e., 5-Azacytidine, zebularine) or non-nucleoside analogs (i.e., hydralazine) against DNA methylation or short fatty acids, hydroxy-cinnamic acids, cyclic tetrapeptides and benzamide against histone deacethylases [180,181]. Notably, a disadvantage of the drugs that act against epigenetic modification consists in lack of specificity. However, their systemic administration can activate oncogenes, which are involved in promotion of malignancy [182]. Besides the epigenetic inhibitors used to overcome drug resistance, Baylin proposed a mechanism based on withdrawal of the chronical drug administration, which in turn will reduce the number of cancer cells with epigenetic modifications and will increase the heterogeneity of the tumor cells, making them sensitive to other anticancer therapies [183]. All these studies and challenges make epigenetic alterations attractive candidates for further therapeutic applications. 

### 2.11. Dysregulation of microRNA (miRNAs)

miRNAs are a family of small single-stranded non-coding RNAs of 20–25 nucleotides. Usually, their main function is downregulation of gene expression at post-transcriptional level [184]. The dysregulation of miRNAs in cancer cells can lead to drug resistance by abnormal modulation of genes expression responsible for MDR, such as (i) ABC transporter genes, (ii) genes related to apoptosis and autophagy, (iii) drug metabolism genes, (iv) DNA repair or (iv) redox system relating genes [93,184]. 

Regarding miRNAs role in regulation of MDR transporters, it was shown that downregulation of miR-38 and miR-200c led to doxorubicin resistance in breast cancer cells, through upregulation of BCRP protein [93]. Downregulation of miR-7 led to drug resistance in lung cancer, through upregulation of MRP1 [93]. Upregulation of several miRNA (miR-16, miR-17) sensitize resistant lung cancer cells to paclitaxel treatment through inhibition of beclin 1 and Bcl-2, promoting apoptosis. Moreover, it was shown that downregulation of miR-17-5p sensitizes colorectal cancer cells to chemotherapeutic agents (5-FU), through increased activity of PTEN [93]. 

miRNAs are also involved in chemotherapeutic agents metabolism; for example miR-27b negatively regulates CYP1B1 expression, while miR-892a regulates CYP1A1 activity and sensitize cells to a wide spectrum of chemotherapeutic agents [184]. Moreover, it was shown that miR-27a contributes to cisplatin resistance by modulation of GSH biosynthesis [184]. Several miRNAs modulate chemosensitivity of cancer cells through interfering with DNA repair mechanisms. For example, over–expression of miR-21 downregulated the expression of mismatch repair (MMR) proteins, thus reducing the therapeutic effect of 5-FU in colorectal cancer cells [184]. In conclusion, miRNAs can serve as therapeutic agents for overcoming MDR [90].

### 2.12. Modulation of Reactive Oxygen Species (ROS)

Modulating reactive oxygen species (ROS) represent a challenging approach to reverse MDR in cancer cells. It is well known that ROS level and the activity of antioxidant enzymes (glutathione peroxidase—GPX, glutathione-S-transferase, catalase, superoxide-dismutase—SOD, hem-oxygenase 1, NAD(P)H quinone oxidoreductase 1, glutamate/cysteine antiporter solute carrier family 7 member 11—xCT, etc.) in MDR cancer cells are overexpressed compared to non-MDR cells [185,186]. Overexpression of ROS facilitate MDR, through upregulation of different pathways (i.e., MAPK, JNK, Nf-kB, PI3K/AKT, Keap1-Nrf2-ARE) [55,75,185]. According to recent research, cancer cells expressing Nrf2 are resistant to chemotherapeutic agents (doxorubicin, etoposide, cisplatin) by increasing GSH production and upregulation of MRP1 [75]. According to Zeng et al., the transcriptional factor src/STAT3 also promotes MDR in cancer cells by promoting antioxidant feedback, through increased expression of GPX and SOD2 activity [187].

Usually ROS are produced by the highly reactive mitochondrial electron transport chain of aerobic respiration, oxido-reductase enzymes (xanthinoxidase, cyclooxygenase, NADPH oxidases—NOXs, etc.) or metal catalyzed oxidation [185]. Recent research has shown that mitochondrial functions are altered in cancer cells, due to imbalance between fusion/fission dynamics and increased mitophagy, which grants a rapid clearance of chemotherapeutic agents, increases ABC transporters activity (by providing ATP) and modifies mitochondrial membrane potential [75].

Several agents (current in preclinical or clinical studies) are involved in modulation of ROS in MDR by (i) disrupting mitochondrial electron transport chain (elesclomol), (ii) inhibition of NOXs (ampelopsin), (iii) depletion of intracellular GSH (APR246), (iv) inhibition of xCT, required for GSH synthesis (erastin, vorinostat) or (v) inhibition of Nrf2 pathway (camptothecin) [185].

## 3. Role of Polyphenols in MDR

### 3.1. In Vitro Studies 

#### 3.1.1. Flavonoid Compounds

##### Flavones 

Flavonoid compounds were intensively tested for their capacity to enhance the effect of anti-cancer drugs and to combat MDR in different types of cancers. An experiment conducted on CD44^+^ prostate cancer stem cells provided relevant information that *apigenin* co-administrated with cisplatin stimulated the therapeutic effects of cisplatin by inducing a series of modulatory effects on the expression of essential proteins and enzymes [188]. The mechanism of apoptosis induced by tumor necrosis factor-related apoptosis-inducing ligand (TRAIL) was studied in conjunction with flavonoids as potentiating agents, due to the high occurrence of TRAIL resistance in various cancer types. In this regard, Yang et al. demonstrated that *wagonin* showed the capacity to enhance apoptosis mediated by TRAIL in vitro through downregulating the expression levels of anti-apoptotic proteins [41]. 

According to Rao et al., *luteolin* overcomes MDR in breast cancer mitoxantrone resistant cells through increased apoptosis, DNA damage, activation of ATR/Chk2/p53 signaling pathways, inhibition of NF-κB signaling pathway and depletion of anti-apoptotic proteins [189]. 

Nucleoid factor erythroid-2 related factor 2 (Nrf2) is a transcription factor that regulates genes responsible for the synthesis of endogenous antioxidants (hemeoxygenase-1—HO-1), transporters (MRP1, MRP2) and detoxifying enzymes (glutathione-S-transferase) [190]. Recent research have demonstrated that Nrf2 is overexpressed in MDR cancer [190]. According to recent data, co-treatment of breast and lung cancer cells with *luteolin* and chemotherapeutic agents (oxaliplatin, doxorubicin, bleomycin) resulted in a higher percentage of cells death. The suggested mechanisms involve downregulation of *NRF2* gene expression (MDR and HO1) and increased sensitization of the cells to chemotherapeutic treatment [190,191].

##### Flavonols

*Quercetin* was found to suppress effects of P-gp in breast cancer cells and to increase the disappearance of breast cancer stem cells. In this case, doxorubicin-resistant MCF-7 cells were evaluated for how they respond to different drugs (doxorubicin, paclitaxel and vincristine) in conjunction with quercetin. It was found that the co-administration of these drugs with quercetin potentiated their chemotherapeutic effect [192]. The potential of quercetin to reverse the MDR process through the inactivation of P-gp was also revealed on vincristine resistant human colorectal adenocarcinoma Caco-2 cells [193]. Another study performed on Caco-2 cells showed that quercetin as well as naringenin and genistein manifested inhibitory effects on cell elimination of cimetidine through P-gp activity [194]. Downregulation of P-gp by quercetin and other flavonoids such as naringenin, biochanin A, silymarin, genistein was successfully demonstrated in daunomycin resistant MCF-7 breast cancer cell lines. It was shown that these compounds not only stimulated the accumulation of the drug, but also substantially reduced its efflux [195]. 

It was also observed that *fisetin*—another dietary flavonoid compound—changed the MDR course of action leading to the chemosensitizing effects on colorectal cancer cells resistant to common chemotherapeutic drugs. Co-administration of fisetin with irinotecan and oxaliplatin induced apoptosis in cultured cells by increasing the activity of caspase-8 and caspase-3. Furthermore, this combined treatment triggered the efflux of cytochrome C and considerably reduced the phosphorylation mechanisms of IGF1R and AKT [196].

##### Flavanones

*Hesperidin* (hesperitin rutinoside) was able to increase the sensitivity of breast resistant cancer cells to doxorubicin, through decreased expression of P-gp [197]. Moreover, El-Readi M.Z. and his co-workers have shown that hesperidin had a significantly higher inhibitory effect of P-gp than nobiletin and stigmasterol but lower effect than limonin in overcoming MDR in colorectal cancer cells [198].

##### Flavan-3-ols

Impeding DNA damage repair processes through dietary flavonoids was also shown to be a successful endeavor in combating chemoresistance in cancer. It was found that quercetin, *catechin* and fisetin intensified the sensitivity of breast cancer cells to cisplatin by inhibiting ATR-Chk1 pathway [199]. Green tea polyphenols have also shown inhibitory properties towards efflux pumps (P-gp) [200]. The inhibitory effect decreased as follows epigallocatechingallate > epigallocatechin > catechin > epicatechin [201]. EGCG induces the reversal of MDR by regulating detoxification mechanisms and downregulation of Nrf2 pathway in breast cancer cells resistant to tamoxifen [202]. Moreover, according to La X. and co-workers, EGCG enhances the sensitivity of colorectal cancer cells to 5-fluorouracil by inhibiting GRP78/NF-κB/miR-155-5p/MDR1 pathway [203]. Green tea polyphenols (EGCG) associated with quercetin enhanced the therapeutic effect of docetaxel in metastatic and castration-resistant prostate cancer through downregulation of MRP expression, decreased percentage of CD44^+^/CD24^−^ stem-like cells and induced inhibition of PI3K/AKT/STAT3 signaling pathway [204]. Receptor tyrosine kinase signaling pathway has been reported to promote cell proliferation, inhibit apoptosis and to play a major role in MDR. EGCG was shown to reverse MDR in cisplatin resistant lung cancer through downregulation of several receptor tyrosine kinases [205].

##### Isoflavones

*Genistein*—an isoflavone found mainly in soybeans—overcomes chemoresistance to doxorubicin in MDR breast cancer cells through increased accumulation of the chemotherapeutic agent, promotion of apoptosis and suppression of HER2 mRNA expression. However, it had no effect on MDR-1 expression [206]. According to Li and co-workers (2005), genistein pre-treatment of prostate and lung cancer cells inhibits NF-κB activity and contributes to increased growth inhibition and apoptosis induced by cisplatin and docetaxel [207]. Another isoflavone, *daidzein*, found in soybeans, inhibited BCRP and MRP1/2 drug transporters, therefore sensitizing breast cancer cells to chemotherapeutic agents (mitoxantrone, doxorubicin) [208].

#### 3.1.2. Non-Flavonoid Compounds

##### Stilbenes 

*Resveratrol* is a polyphenol commonly found in red wine and grapes that possesses strong antioxidant and anti-aging properties [209]. According to several studies co-administration of resveratrol and other therapeutic agents (paclitaxel, docetaxel, doxorubicin, rapamycin, gefitinib) reversed MDR in breast, lung and colorectal cancer through enhancement of chemotherapeutic agents bioavailability, increase drug retention time, stimulation of pro-apoptosis mechanisms, cell cycle arrest or downregulation of ABC transporters [209,210,211,212,213,214]. 

##### Lignans 

Co-encapsulation of *honokiol* (a lignan isolated from the bark, stem and leaves of *Magnolia* sp.) and paclitaxel in pH-sensitive polymeric micelles suppressed MDR in breast cancer through downregulation of P-gp expression and increase of plasma membrane fluidity [43]. Moreover, honokiol radiosensitizes colorectal cancer cells due to higher levels of apoptosis (caspase-3 activation, increased Bax/Bcl-2 ratio) and reduced expression of cyclin A1 and D1 [215]. 

Other lignans, such as *schizandrin A,* isolated from *Schisandra chinensis* fruits enhanced chemosensitivity of colorectal carcinoma cells to 5-FU through upregulation of miR-195. In addition, upregulation of miR-195 inactivated NF-κB and PI3K/AKT signaling pathways [48]. *Silybin* is the major active constituent of silymarin (a mixture of flavonolignans) from milk thistle fruits. According to Molavi et al., silybin treatment of breast cancer cells resistant to doxorubicin/paclitaxel, sensitized cells to chemotherapeutic agents by suppressing the key oncogenic pathways STAT3, AKT and ERK [46]. According to recent research, a combination of flaxseed lignan (*secoisolariciresinol*) and its metabolite (*enterolactone*) enhanced the cytotoxic effects of docetaxel, carboplatin and doxorubicin in metastatic breast cancer cell lines, likely by inhibition of fatty acid synthase [47].

##### Ellagitannins

Ellagitannins and their metabolite, *ellagic* acid, overcome MDR in cancer, by inhibition of P-gp, MRP and BCRP proteins [216]. Ellagic acid sensitizes human colorectal cancer cells to 5-FU treatment through increased Bax/Bcl-2 ratio, activation of caspase-3 and loss of mitochondrial potential [217]. Ellagitannins and their metabolites play a key role for overcoming MDR in breast resistant cancer cell line [218]. Berdowska et al. have studied the effect of several ellagitannins (agrimoniin, sanguiin-H6, tellimagrandin I, rugosins A, D and pedunculagin) on doxorubicin-resistant breast cancer cells. Among the tested compounds, only sanguiin-H6 showed cytotoxic effects towards resistant MCF-7 cancer cells, probably due to the release of sanguisorbic acid dilactone, which inhibited ABC transporters, thus diminishing the ability of cells to extrude other products of sanguiin-H6 hydrolysis (ellagic acid, depsides), with cytotoxic effects [218]. 

##### Hydroxy-Benzoic Acids

Among phenolcarboxylic acids, *gallic acid* induces apoptosis, enhances the anticancer effect of cisplatin in human lung cancer and reverse MDR [219]. Mechanisms responsible for above-mentioned effects include induction of apoptosis by ROS generation, disruption of mitochondrial membrane potential, increase in the expression of Bax, APAF1, DIABLO and p53 and decrease in the expression of inhibitor of apoptosis protein 3 [219]. In addition, association between gallic acid and ECGC attenuated MDR in doxorubicin-resistant breast cancer cells through a concentration-dependent inhibition of metalloproteinases (MMP-2 and MMP-9). It is well known that metalloproteinases are involved in the degradation of extracellular matrix by metastatic cancer cells [220]. Another mechanism involved in gallic acid overcoming MDR is the inhibition of Src/STAT3-mediated signaling and the decrease in the expression of STAT3-regulated tumor-promoting genes, therefore inducing apoptosis and cell cycle arrest. It is well known that activation of STAT3 signaling pathway is associated with resistance to tyrosine kinase inhibitors, which are frequently used in lung cancer treatment [221]. 

##### Hydroxy-Cinnamic Acids

*Ferulic acid* and *caffeic acid* isolated from foxtail millet (a Chinese cereal food) reverse MDR in human colorectal cancer cells through decreased expression of MRP1, P-gp and BRCP [222]. 

*Caffeic acid phenetyl ester (CAPE)* is a strong inhibitor of human breast cancer stem cells by inhibition of cells’ renewal, progenitor formation and decrease in CD44^+^ cells content. CD44^+^ cells are responsible for tumor formation from a very few cells and are resistant to chemotherapy [223]. According to Khoram et al., CAPE augments the radio sensibility of breast cancer cells [224]. Moreover, CAPE shows beneficial effect in overcoming MDR in lung and prostate cancer through depleting intracellular stores of GSH (reduced glutathione), blocking NF-κB pathway, downregulation of apoptosis inhibitors (cIAP1, cIAP-2 and XIAP) and claudin-2 expression [225,226]. According to recent research, treatment of lung adenocarcinoma derived stem-like cells with *cinnamic acid* diminishes their proliferation and facilitates their differentiation into CD133 (a marker used for isolation of cancer stem cell population mainly from carcinomas) negative cells [227]. 

##### Other Compounds

*Curcumin* is the major active substance of the culinary spice turmeric (*Curcuma longa*) and has strong antioxidant, anti-inflammatory and anti-cancer effects [34,50,228,229]. Curcumin has been reported to attenuate oxaliplatin and 5-fluorouracil (5-FU) acquired resistance in colorectal and breast cancer cells through inhibition of NF-κB signaling cascade [230,231]. Moreover, association between curcumin and oxaliplatin downregulated the expression of NF-κB regulated gene products involved in inflammation (CXC-chemokines, which are highly overexpressed due to acquired resistance) and decreased the levels of p65 [230]. Recent research has shown that a curcumin-derivative (difluorinated curcumin) inhibits 5-FU and oxaliplatin resistant colorectal cancer cells through downregulation of miR-21. miR-21 downregulates PTEN, a tumor suppressor gene. Decreased activity of PTEN is involved in resistance to conventional therapy and recurrence of cancer initial treatment [232]. Moreover, PTEN downregulates Nrf2 activity and autophagy, which have been reported to play a protective role in cisplatin induced apoptotic cell death [233]. According to Gu et al., nanomicelles loaded with doxorubicin and curcumin alleviate MDR in lung cancer, due to increased cellular uptake of chemotherapeutic agents [234]. According to recent studies, curcumin reverses cisplatin resistance and promotes human lung adenocarcinoma apoptosis through increased apoptosis and down-regulation of HIF-1α [235]. It has been shown that curcumin inhibits mammalian target of rapamycin (mTOR)—a serin/threonine kinase—and downregulates the key epigenetic regulator enhancer of zeste homolog 2 (EZH2) in tamoxifen resistant breast cancer cells [236]. According to Thulasiraman, curcumin also restores sensitivity to retinoic acid in triple negative breast cancer cells by suppressing the expression level of fatty acid-binding protein 5 (FBAP5) and peroxisome proliferator-activated receptor β/δ (PPARβ/δ) [237]. The combination of curcumin with other phenolic compounds (such as EGCG) showed synergistic effects in overcoming doxorubicin-resistant tumor breast cells through caspase-dependent apoptotic signaling pathways, downregulation of anti-apoptotic Bcl-2 and survivin, and enhancement of cellular incorporation of curcumin [238].

*Gingerol* represents the main active substance from dry or fresh ginger roots, a popular spice widely used in many diseases (nausea, diarrhea and cancer) [51]. According to Liu Chin-Ming and co-workers, 6-gingerol and 10-gingerol inhibited the proliferation of docetaxel resistant human prostate cancer cells through downregulation of MRP1 and GST [51]. According to recent research, 6-gingerol shows high anticancer potency in cyclophosphamide, 5-FU and doxorubicin-resistant breast cancer MCF-7 cell line, due to its antioxidant activity and regulation of different cellular pathways (Wnt-β catenin or glycogen synthase kinase 3—GSK3) [239].

In conclusion, recent in vitro studies (Table 2) have shown that phenolic compounds overcome MDR in different types of cancer (breast, lung, prostate, colorectal) by inhibition of efflux pumps (P-gp, MRP1, BCRP), increased apoptosis and decreased proliferation of cancer stem cells, increased cellular uptake of chemotherapeutic agents, downregulation of miR-27a, miR-195, miR-21, inactivation of DNA damage repair, decreased expression of anti-apoptotic proteins and modulation of important signaling pathways involved in carcinogenesis (PI3/Akt, Wnt-β catenin, GSK-3, NF-κB, mTOR, Nrf2, ERK, JNK, etc.). 

Considering the evidence provided by in vitro studies, continuous pharmacological research (pre-clinical and clinical studies) is needed in order to verify the potential beneficial effects of polyphenols in vivo and to discover new mechanisms of action for overcoming MDR.

#### 3.1.3. Synergic and Pleiotropic Activity of Polyphenols 

Recent data support the hypothesis that combined drug therapy might be more efficient than monotherapy (“one drug-one target” therapy). The synergistic effects of combined administration of polyphenols appears mainly at a molecular level, since they influence different pathways involved in multidrug resistance. For example, association between curcumin and EGCG showed synergistic effect in overcoming doxorubicin resistance in tumor breast cancer cells [238]. The synergistic effect occurs due to inhibition of P-gp expression by EGCG, thus increasing the incorporation of curcumin in breast cancer cells, leading to enhancement of apoptosis and regulation of apoptosis proteins [238]. A similar effect was observed for the association between EGCG and gallic acid in multidrug resistant MCF7/DOX breast cancer cells [220]. The inhibitory effect of EGCG upon P-gp increases gallic acid concentration in cancer cells leading to inhibition of matrix metaloproteinases (MMP-2, MMP-9). Regarding the combination of EGCG and quercetin in docetaxel resistant prostate cancer cells [204], both compounds are strong inhibitors of P-gp [240]. Consequently, both compounds have increased concentrations in prostate cancer cells and act by inhibition of PI3K/AKT, STAT3 signaling pathways and decreased cancer stem cells activity [204]. Since the data regarding the interactions between polyphenols in MDR models are promising but limited, this might represent starting points for future studies. 

The pleiotropic effect of the polyphenols has already been acknowledged in the scientific publications [241,242]. Based on the reported data, polyphenols overcome multidrug resistance by affecting different pathways in different types of cancer [243]. For example: (i) quercetin increases apoptosis, inhibits angiogenesis (in colorectal cancer cells) [244], inhibits P-gp activity (in breast cancer cells) [245]; (ii) curcumin down-regulates P-gp and Hsp27, induces autophagy, reduces the markers of cancer stem cells (colon cancer cells) [246,247,248], inhibits the activity of ABCB4 pump, inhibits epithelial-mesenchymal transition (breast cancer cells) [249,250], inhibits JNK pathway, suppresses invasion by inhibition of STAT3 activity (prostate cancer) [251,252] or induces apoptosis (lung cancer cells) [253]; (iii) resveratrol down-regulates the expression of survivin (in prostate cancer cells) [254] and inhibits MAPK kinase in prostate and lung cancer cells [255]; (iv) EGCG inhibits drug efflux (in prostate cancer cells), increases drug concentration in cancer cells by inhibition of enzymes involved in drug metabolism (in colorectal cancer cells), increased ROS production (in colorectal cancer cells)—thus it is responsible for AMPK activation—and induces epigenetic restoration of estrogen receptors through histone modifications (in breast cancer cells) [256]. Nevertheless, based on reported data, some polyphenols can target the same molecule in different cancer cell lines. For instance, resveratrol can downregulate P-gp in breast, lung and colorectal cancer cells [210,211,212]. Taken together these data suggest that polyphenols are able to modulate different signaling pathways being cell-line-specific and to target certain molecules independent of cell type (Table 2).

### 3.2. In Vivo and Clinical Studies 

#### 3.2.1. Flavonoid Compounds

##### Flavones and Flavonols 

Shin et al. published a study centered on the co-administration of tamoxifen with *quercetin* in rats, showing great evidence of the inhibition of P-gp, MRP2 and BCPR, as well as relevant data, which support the antioxidant property of quercetin through the reduction of CYP3A4 activity [257]. Experiments on animal models confirm the suppressing function of *quercetin* on ABC proteins involved in MDR. 

Co-encapsulation of *quercetin* and doxorubicin in biotin receptor-targeting nanoparticles was more effectively taken up with less efflux due to downregulation of P-gp expression in nude mice bearing MCF-7 breast cancer cells resistant to adriamycin (doxorubicin) [258]. According to et al., applying *wogonin* and TRAIL in a mouse model of lung cancer enhances TRAIL’s antitumor activity and overcomes MDR through augmentation of apoptosis and decreased the expression of anti-apoptotic proteins (survivin, XIAP, etc.) [41]. 

*Fisetin* showed promising effects in a mouse model of lung cancer and prevented MDR through increased apoptosis and downregulation of AKT and IGFR1 phosphorylation levels [196].

*Luteolin*, another flavonoid, was analyzed for its potential beneficial role in reversing MDR in cancer. For this purpose, a group of researchers took into consideration the analysis of xenograft tumors of lung cancer, which were treated with luteolin, erlotinib and cisplatin for 15 days. They concluded that the group of mice treated with luteolin and cisplatin showed the most relevant reduction in the tumor mass. Moreover, luteolin was shown to sensitize tumor cells to erlotinib through downregulation of EGFR/PI3K/AKT/mTOR signaling pathway and increased apoptosis [259].

##### Flavan-3-ols

Combining *EGCG* with paclitaxel induced significant cell apoptosis in a murine model of breast carcinoma. Moreover, EGCG overcame MDR to paclitaxel by inhibiting GRP78 expression and inhibition of JNK phosphorylation [260]. In a rat model of breast carcinogenesis application of EGCG overcame MDR to paclitaxel through increased apoptosis, decrease of cancer stem cells, decreased VEGF expression and MMP-2 activity [261].

##### Isoflavones 

The potential of *genistein* to cause inhibition of MDR in lung cancer was intensively studied. One representative case is the assessment of the genistein-cisplatin treatment of non-small cell lung cancer (NSCLC) in xenografted mice models, in order to prove the sensitization of drug-resistant cancer cells via enhanced activity of caspase-3, 8, 10 and suppression of PI3K/AKT activity [262]. The property of genistein to sensitize NSCLC cells was demonstrated for another chemotherapeutic agent, gefitinib. In this respect, it was acknowledged that the combinatory treatment using genistein and gefitinib increased apoptosis and downregulated EGFR and mTOR signaling pathways [263].

#### 3.2.2. Non-Flavonoid Compounds

##### Stilbenes

Co-encapsulation of resveratrol and paclitaxel in a PEGylated liposome showed effective inhibitory effects in drug-resistant breast tumors in mice through increased cellular uptake of paclitaxel and decreased activity of efflux pumps (MRP, P-gp) [264]. According to Yang et al., resveratrol sensitized colorectal cancer cells to oxaliplatin, mainly by upregulation of miR-34c in correlation with increased levels of p53 and reduction of tumor growth in xenograft experiments [265]. Resveratrol significantly inhibited MDR in nude mouse models inoculated with human non-small cell lung cancer cells by downregulation of survivin and activation of caspase-3 [266].

##### Hydroxy-Cinammic Acids

*Caffeic acid phenethyl ester* (CAPE) reverses MDR in breast cancer mouse models due to downregulation of anti-apoptotic and cell proliferation genes, as well as NF-κB transcription factors. Moreover, it decreased *MDR1*-gene expression, so it might be used as an adjuvant to chemotherapeutic agents (paclitaxel) treatment [267].

##### Lignans

*Podophyllotoxin*, a lignan, found in the roots of *Podophyllum peltatum* L. exhibited significant activity against P-gp mediated MDR tumor cell lines [44]. However, due to its poor solubility, it cannot be used systemically. Nanoparticles composed of poldophyllotoxin and polyethylene glycol with acetylated carboxymethyl cellulose showed beneficial effects in breast and prostate resistant tumor models in mice through enhanced sensitization of tumor cells to chemotherapeutic agents and increased tumor penetration [44]. Moreover, the delivery of nanoparticles was highly selective to the tumors with minimal uptake in other tissues [44]. Another lignan, *deoxypodophyllotoxin* from the roots of *Anthriscus sylvestris* exhibited better efficacy to MDR in mouse models for breast cancer than paclitaxel [45]. According to Lou S. and co-workers a multifunctional nanosystem composed of doxorubicin, paclitaxel and *silybin* controlled drug release, decreased P-gp activity and synergistically inhibited breast tumors growth [268].

##### Other Compounds

In vivo studies have shown that *curcumin* sensitizes human colorectal cancer to capecitabine in an orthotopic mouse model, through inhibition of NF-κB, decreased expression of genes enconding for proteins involved in proliferation (COX-2), invasion (MMP-2, ICAM-1), metastasis (CXCR4), angiogenesis (VEGF) and anti-apoptotic gene products (Bcl-2, IAP-1 and survivin) [269]. Other authors reported that curcumin regulates colorectal cancer by inhibiting P-gp in in situ cancerous colon perfusion in a rat model. Inhibition of P-gp enhanced the cytotoxic effects of irinotecan [270]. According to Howells L. and co-workers curcumin also ameliorates oxaliplatin-induced chemoresistance in HCT-116 xenograft tumors by preventing oxaliplatin-induced upregulation of ALDH1 and decreased activity of excision nucleases, by which DNA lesions are repaired [271]. Administration of nanoparticles with docetaxel/doxorubicin and curcumin to mice inoculated with prostate cancer cells, overcame MDR to chemotherapeutic agents through enhanced cellular uptake of chemotherapeutic agents and inhibition of MDR1 and MRP [272,273]. Moreover, it was shown that curcumin decreases doxorubicin cardiotoxicity [273]. Besides, curcumin chemosensitizes prostate cancer cells to gemcitabine by downregulation of MDM2 oncogene through PI3K/mTOR/ETS2 pathway [274]. Cheng et al. investigated the effect of co-administration of curcumin and phospho-sulindac in a mouse xenograft model of human lung cancer. The results were promising, with improved phospho-sulindac pharmacokinetics and higher levels of the chemotherapeutic agent and its metabolites in the xenografts. It was observed that curcumin enhances phospho-sulindac accumulation in cancer tissues through inhibition of P-gp and MRPs [275]. Cui et al. demonstrated that administration of nanoparticles containing a pH-sensitive pro-drug transferrin-poly(ethylene glycol)-curcumin and doxorubicin exhibited higher cytotoxicity and sensitivity in breast cancer xenograft mouse model compared to the chemotherapeutic agent alone [276]. 

Few studies have investigated the effect of phenolic compounds for overcoming MDR in humans. According to Mahammedi et al., the combination of curcumin with docetaxel and prednisone showed a high-response rate, good tolerability and acceptability by patients with castration-resistant prostate cancer. It was shown that curcumin reverses docetaxel induced NF-κB activation [277]. Association between curcumin and docetaxel showed beneficial effects in women with advanced and metastatic breast cancer. Curcumin/docetaxel combination demonstrated significant anti-tumor activity, decreased levels of VEGF and other angiogenic growth factors (TGF-α). Moreover, curcumin improved docetaxel bioavailability and reversed drug resistance through downregulation of P-gp expression [278]. 

Taken together, these results shown that phenolic compounds overcome MDR in different types of solid cancer (breast, lung, prostate, colorectal) both in vivo and in clinical studies (Table 3). However, the data regarding clinical studies with polyphenols and multidrug resistance are very scarce. The mechanisms are generally the same, as previously reported for in vitro studies.

#### 3.2.3. Bioavailability and Toxicity of the Polyphenols

Although several studies have shown the beneficial effects of some plant polyphenols in overcoming multi-drug resistance in breast, colorectal, lung, prostate, most of the research was performed using only in vitro (cell lines) and in vivo (animal) models. However, data regarding clinical studies with polyphenols for overcoming chemoresistance are scarce. The extrapolation of the results from pre-clinical studies to humans is difficult and risky, keeping in mind that polyphenols bioavailability is complex and influenced by several factors: (i) chemical structure, (ii) liberation from the food/medicinal plant matrix, (iii) gastro-intestinal absorption, (iv) metabolism by gut microbiota, liver, enterocytes, (v) plasma transport, plasma concentration, (vi) distribution and elimination [40,279,280,281]. Polyphenols bioavailability is relatively low, due to low absorption, extensive biotransformation and rapid clearance from the body [281]. Still, polyphenols metabolites (produced by gut microbiota or liver) reach higher plasma concentrations compared to their parent compounds are considered responsible for polyphenols therapeutic effects. Several polyphenols metabolites such as urolithins (ellagitannins metabolites), enterolactone and enterodiol (lignans metabolites), equol (isoflavones metabolite) have shown a chemopreventive role in breast, prostate or colorectal cancer [282,283]. Taken together, clinical studies are imperative in order to demonstrate the beneficial role of polyphenols in overcoming multidrug resistance in various types of cancer.

In spite of promising results from laboratory experiments, implementation of them into the clinical trials might represent a challenge due to higher concentrations used in those studies. Nevertheless, several clinical studies validated the efficiency of polyphenols against different types of solid tumors [284,285,286]. Administration of regular cytostatic drugs is correlated with severe side effects, such as bone marrow modifications (leucopenia, thrombocytopenia, anemia), nausea, vomiting, alopecia, drug extravasation, hepatotoxicity or heart toxicity [287,288]. Conversely, the polyphenols toxicity is greatly reduced and the side effects could be constipation/diarrhea, dry mouth or flatulence [289]. For example, association of curcumin (0.5, 1, 2 g) for seven days prior to FOLFOX (5-fluorouracil, oxaliplatin, folinic acid) chemotheraphy (two-weekly cycles to a maximum of 12 cycles) in patients with colorectal cancer and liver metastasis, led to several side effects. The most common side effects, which were related to curcumin use (not with FOLFOX) were constipation, dry mouth and flatulence. One patient reported severe diarrhea, attributed to curcumin. Diarrhea was treated when curcumin dosage was changed from 2 g to 1 g and the dosage change did not affect the anticancer effect of curcumin [289].

As general considerations, if any of the cytotoxic effects are visible it is recommended to stop the treatment before the irreversible toxic effects occur. In addition, for better toleration of the treatment it is recommended to start the administration when the patient is in good physical condition [290]. Several general recommendations might be taken in account to reduce toxicity of the polyphenols:

(i) Combinatorial treatment. Administration of more than one polyphenols or the use of polyphenols as adjuvants in chemotherapy might reduce the concentration of the polyphenols when administrated. For instance, in human colon cancer cells with P-gp overexpression the synergism between DOX and EGCG/curcumin was demonstrated. Thus, lower concentration of DOX and polyphenols are required when co-administrated compared to single drug administration [291]. Similar synergism was seen in human colorectal cells treated with platinum-based compounds, such as oxaliplatin, cisplatin and EGCG [292]. 

(ii) Replacement of the natural compound with another one. In a clinical study performed in 49 patients with solid tumors (non-small cell lung cancer, head and neck cancer) the administration of capsules containing a green tea extract (GTE) (standardized in 26.9% total catechins – EGCG – 13.2%; epicatechin 2.2%; epicatechin gallate 3.3%; epigallocatechin 8.3% and 7% caffeine), at increasing dosages up to 8–10 g GTE once daily or 10–13 g distributed over three daily dosages for minimum four weeks to six months, several side effects occurred: nausea, abdominal bloating, headache, insomnia, tremor and palpitations. It was concluded that caffeine was responsible for the above-mentioned side effects. A possible solution to remedy these adverse effects would be the use of Polyphenon E (which is a decaffeinated GTE standardized in 65% EGCG), which was considered safe, when it was given to chronic lymphocytic leukemia patients (400–2000 mg orally twice a day) for one month [293,294]. However, Polyphenon E should be administered only with food and not after an overnight fast, due to higher EGCG plasma C_max_ (seven-fold higher compared to EGCG administration with food) and high risk of hepatotoxicity [295]. Another polyphenols, resveratrol has shown kidney toxicity in clinical trials. According to Popat and co-workers the administration of a SRT501, a micronized oral formulation with resveratrol (5 g/day for 20 days in a 21 days cycle, up to 12 cycles followed by bortezomib) in patients with relapsed or refractory multiple myeloma, led to severe side effects (renal failure, nausea, anemia etc.). Renal failure occurred within the first two cycles of SRT501 monotherapy. However, it seems that SRT501 induces kidney failure only in myeloma patients, since the same dose of SRT501 was safe in diabetic patients or stroke-like episodes syndrome [296]. A solution to remedy renal failure in myeloma patients is the administration of a grape seed extract (rich in resveratrol but also other phenolic compounds. i.e. quercetin, proanthocyanidins), that have strong antioxidant effects and are able to protect the kidneys [297].

(iii) Validation the purity of the natural compound. The administration of a green tea extract (rich in catechins, mainly epigalocatechin gallate 11.8–4509 mcg/g extract), in a dosage of 5.9 g over five days to 240 g over 120 days was responsible for hepatic toxicity, mainly acute hepatocellular injury. Still, patients fully recovered with drug cessation [298,299]. According to some authors the observed hepatic toxicity of green tea extracts might be the consequence of contamination with pesticides (endosulfan), which is extensively used in green tea plantations [300].

(iv) Modes and route of administration. To increase specificity of polyphenols, they can be administrated as nanoparticles which have been coated with antibodies directed against molecular markers from the surface of the tumors [301,302]. In addition, local administration of the compound might be used whenever possible [301].

## 4. Conclusions

MDR has become the most important obstacle to the success of cancer chemotherapies. It implies several mechanisms, such as increased activity of efflux pumps (MRP 1/2, P-gp, BCRP), inhibition of cell death, cancer stem cells, epigenetic mechanisms, increased DNA repair, modification of drug target, inactivation of anticancer drugs, tumor cell heterogeneity, tumor microenvironment and epithelial to mesenchymal transition. 

The use of natural compounds could overcome MDR through various mechanisms. Several studies have been performed using flavonoid (apigenin, luteolin, quercetin, genistein, epigallocatechin gallate, etc.) and non-flavonoid compounds (lignans, gallic acid, resveratrol, curcumin, etc.). In vitro and in vivo studies have revealed that administration of polyphenols (both from dietary sources and medicinal plants) overcome MDR to chemotherapeutic agents (paclitaxel, 5-fluorouracil, docetaxel, doxorubicin, gefitinib, etc.) in different types of cancer (breast, lung, prostate and colorectal) by downregulation of efflux pumps and anti-apoptotic proteins (survivin, XIAP), downregulation of NF-κB signaling cascade, decreased stem cells progenitor formation, increased cellular uptake of chemotherapeutic agents, epigenetic mechanisms, upregulation of apoptotic factors (DIABLO, APAF1) or modulation of several signaling pathways (Sonic-Hedgehog, EZH2, HER2, ERK, JNK, PI3K/AKT, STAT3, Wnt/β-catenin, etc.) and enzymes (FAS, GSK3, MMP2/MMP9, GST, etc.). However, few clinical studies demonstrated these effects. Therefore, we hope that this review will lead to continuous research regarding the role of phenolic compounds in overcoming multidrug resistance in various types of cancer.

## Figures and Tables

**Table 1:** Main classes of phenolic compounds with representative members and sources, frequently investigated for overcoming MDR in cancer.

Phenolic Compounds	Chemical Structure	Representative Compounds	Sources	Reference
**Flavonoid Compounds**				
Flavones	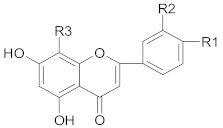	apigenin (R_1_–OH, R_2_–H, R_3_–H) luteolin (R_1_–OH, R_2_–OH, R_3_–H) wogonin (R_1_–H, R_2_–H, R_3_–OCH_3_)	oranges, lemons, apricots, apples, black currants, bananas, potatoes, spinach, onions, lettuce, parsley, celery, beans, tomatoes, roots of *Scutellaria baicalensis* Georgi	[37,38,40,41]
Flavonols	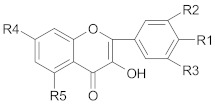	quercetin (R_1_–OH, R_2_–OH, R_3_–H, R_4_–OH, R_5_–OH) fisetin (R_1_–OH, R_2_–H, R_3_–OH, R_4_–OH, R_5_–H)		
Flavanones	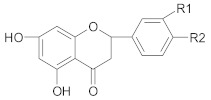	naringenin (R_1_–H, R_2_–OH) hesperitin (R_1_–OCH_3_, R_2_–OH)	oranges, grapefruits, lemons	[40]
Flavan-3-ols	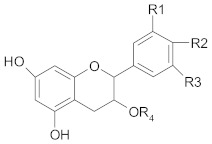	catechin (C), epicatechin (EC) (R_1_–OH, R_2_–OH, R_3_–H, R_4_–H) epigallocatechin (EGC) (R_1_– OH, R_2_–OH, R_3_–OH, R_4_–H) epigallocatechingallate (EGCG) (R_1_–OH, R_2_–OH, R_3_–OH, R_4_— 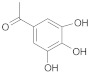 )	green/black tea, grapes, cherries, apricots, peaches	[38,40]
Isoflavones	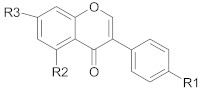	genistein (R_1_–OH, R_2_–OH, R_3_–OH) daidzein (R_1_–OH, R_2_–H, R_3_–OH)	soy flour, soy paste (natto, cheonggukang), soy bean (roasted)	[38]
**Non-Flavonoid Compounds**				
Hydroxy-benzoic acids	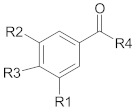	gallic acid (R_1_–OH, R_2_–OH, R_3_–OH, R_4_–OH)	blackcurrants, strawberries, raspberries, kiwi, cherry, plums, spinach, broccoli	[40,42]
Hydroxy-cinnamic acids	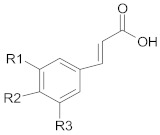	caffeic acid (R_1_–H, R_2_–OH, R_3_–OH) ferulic acid (R_1_–H, R_2_–OH, R_3_–OCH_3_) cinnamic acid (R_1_–H, R_2_–H, R_3_–H)	plums, apples, eggplants, potatoes, wheat, rice, oat, kiwi	[40]
	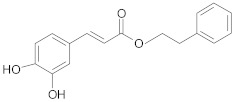	caffeic acid phenethyl ester (CAPE)	artichoke, oregano, thyme, basil, coffee, mushrooms	[40]
**Lignans**	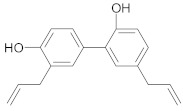	honokiol	bark, root, seeds, leaves of *Magnolia* sp.	[43]
	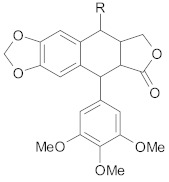	podophyllotoxin (R–OH) deoxypodophyllotoxin (R–H)	rhizome of American mayapple (*Podophyllum peltatum* L.) roots of *Anthriscus sylvestris* L. (Hoffm.)	[44,45]
	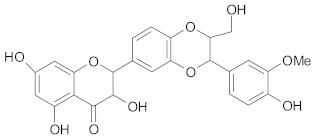	silybin (silibinin)	fruits of milk twistle (*Silybum marianum* L.) Gaerth	[46]
	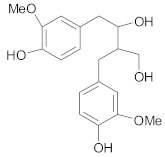	secoisolariciresinol	flaxseeds	[47]
	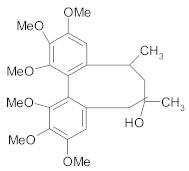	schizandrin A	fruits of *Schisandra chinensis*	[48]
**Ellagitannins**	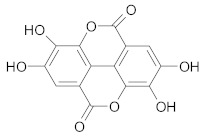	ellagic acid	raspberries, strawberries, pomegranate black currants, blackberries	[49]
	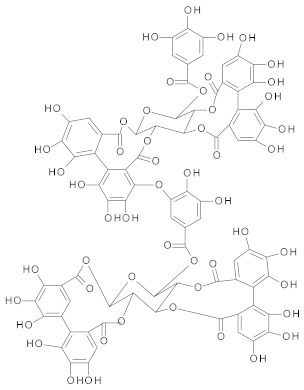	sanguiin-H6	raspberries	[49]
**Stilbenes**	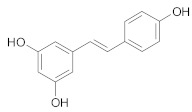	resveratrol	grapes, mulberries	[40]
**Other Compounds**	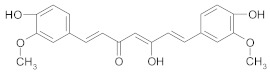	curcumin	*Curcuma* roots	[50]
	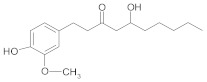	gingerol	fresh/dried ginger rhizomes	[51]

**Table 2:** Summary of in vitro experiments.

Compound	Type of Cancer	Cell Line	Treatment/Duration	Mechanisms of Overcoming MDR	Reference
**Flavonoid Compounds**					
Apigenin	Prostate	CD44^+^ PC3 cells	15 μM apigenin + 7.5 μM CDPP, 48 h	↓ Bcl-2, ↓ sharpin, ↓ survivin, ↑ caspase 8, ↑ APAF-1, ↑ p53 mRNA, ↓ NF-κB, ↑ p21, ↓ CDK-2, ↓ CDK-4, ↓ CDK-6	[188]
Wogonin	Lung	A549 cell line	10 μM wagonin + TRAIL (5–20 ng/mL), 24 h	↑ apoptosis, ↓ cFLIP_L_, ↓ XIAP, ↓ cIAP-1, ↓ IAP-2	[41]
Luteolin	Breast	ABCG2 expressing MCF-7 cells mitoxantrone resistant	12.5–100 μM luteolin + 1 μM mitoxantrone, 4 h	↑ ROS, ↑ DNA damage, ↓ NF-κB ↓ cIAP-1, ↓ survivin, ↓ XIAP ↑ ATR-CHk2-p53	[189]
	Breast	MDA-MB 231 cells DOX resistant	5–20 μM luteolin + 0.08–20 mM DOX, 24 h	↓ Nrf2	[191]
	Lung	A549 cells	Pre-treatment (24 h) with 5 μM luteolin before DOX (0–3 μg/mL), OX (0–100 μM), bleomycin (0–100 μM), 48 h	↓ Nrf2	[190]
Quercetin	Breast	DOX resistant MCF-7 cells	2.5 μg/mL DOX, 0.5 μg/mL PTX, 0.5 μg/mL VCR + 0.5. μg/ml quercetin - 24 h	↓ P-gp, ↓ YB-1 nuclear protein translocation, ↓ BCSCs phenotype CD44^+^/CD24^−^/^low^, ↑ apoptosis, cell cycle arrest	[192]
	Colorectal	VCR resistant Caco-2 cells	0.5–200 μM quercetin, 24 h	↓ P-gp	[193]
	Colorectal	Caco-2 cells	20 μM cimetidine + 100 μM quercetin, 4 h	↓ P-gp	[194]
Fisetin	Colorectal	OX-resistant LoVo cells CPT11-resistant LoVo cells	0 μM, 40 μM, 80 μM fisetin, 24 h	↑ apoptosis, ↑ cytochrome C release, ↓ IGF-1R and AKT phosphorylation levels	[196]
Naringenin	Breast	Daunomycin resistant MCF-7 cells	9 × 10^−8^ M– 7.2 × 10^−5^ M daunomycin + 50 μM naringenin, 72 h	↓ P-gp	[195]
Hesperitin glycoside (hesperidin)	Breast	MCF-7 DOX resistant cells	0.5–3.5 μM/L hesperidin + 35–233 nM/L DOX, 24 h	↓ P-gp	[197]
	Colorectal	Coco-2 cells overexpressing P-gp	32 μM hesperidin, 24 h	↓ P-gp	[198]
Catechin	Breast	MDA-MDB-231 CDPP resistant cells	5, 10, 20, 40 μM C + 10 μM CDPP, 6 h	↓ ATR-Chk1 pathway	[199]
EGCG	Breast	Tamoxifen-resistant MCF-7	Nrf2-RNA transfection, 48 h + 50/100 μM EGCG, 24 h	↓ Nrf2 signaling pathway	[202]
	Colorectal	HCT-116 DLD1 cells	50 μM EGCG + 0–30 μM 5-FU, 24 h	↓ GRP78/ NF-κB/miR-155-5p/MDR1 pathway	[203]
	Prostate	PC3, LAPC4 cells	40 μM EGCG + 5 μM quercetin + 5 nM DOC, 24/48 h	↓ CD44^+^/CD24^−^ cells, ↓ MRP1, ↓ PI3K/AKT/ STAT3	[204]
	Lung	A549/H460 CDPP resistant cells	80 μM EGCG + 0–30 μM CDPP, 24 h	↓ Axl, Tyro3	[205]
Genistein	Breast	MCF-7 DOX resistant cells	0–120 μmol/L genistein + 0.7–70 μM DOX, 48 h	↓ HER 2/neu, ↑ apoptosis	[206]
	Prostate Lung	PC-3 cells H460 cells	pre-treatment with 15–30 μmol/L genistein, 24 h 1–2 nM DOC/100 nM/L cisplatin, 48 h	↑ apoptosis, ↓ NF-κB	[207]
Daidzein	Breast	MCF-7/ MDA-MB 231 cells	pre-treatment with 10 μM daidzein, 24 h before administration of 0–10 mM DOX/ mitoxantrone	↓ MRP1/2,↓BCRP	[208]
NON-FLAVONOID COMPOUNDS					
Resveratrol	Breast	MCF-7 cells	100 µM RES + 20 nM rapamycin, 24 h	↓ mTOR, ↓ AKT, ↑ autophagy	[209]
	Breast	DOX resistant MCF-7	4–16 µM RES + 4–64 µM DOX, 24 h	↓ P-gp	[210]
	Breast	SK-BR-3, MCF7, MDA-MB-231, T47D cells	15 µM RES + 1 nM DOC	↓ HER2-AKT axis	[214]
	Lung	NCI-H460 cells	0–20 µg/mL RES + 0–10 µg/mL PTX, 24 h	↓ P-gp, MRP2, BCRP	[211]
	Lung	GF resistant NSCLC- PC9	1–20 µM GF + 5–160 µM RES	↑ apoptosis, ↑ senescence	[213]
	Colorectal	HCT 116, HT-29 cells	0.3 µM DOX + 100 µM RES	↓ P-gp, ↑ Bax, cell cycle arrest	[212]
Honokiol	Breast	MCF-7/DOX, MDA-MB-231	200 µL polymeric micelles with 1 mg PTX + 0.5 mg/L HNK, 24/36 h	↓ P-gp, ↑ plasma fluidity	[43]
	Colorectal	HCT-116 cells	0–50 μM HNK + 0–5 Gy γ-radiation, 24/48 h	↑ apoptosis, ↓ cyclin A1, D1	[215]
Secoisolarici resinol	Breast	MDA-MB-231, SKBR3 cells	25–50 µM SECO, 25–50 µM ENL, 20 nM DOX, 1 nM DOC, 1000 nM CAB, 72 h	↓ FAS	[47]
Schizandrin A	Colorectal	5-FU resistant HCT116, SW-480	0–8 µM 5-FU + 0–40 µM SchA, 48 h	↑ mir-195	[48]
Silybin	Breast	MDA-MB 435 DOX resistant cell line MCF-7 PTX resistant cell line	200–600 μM silybin + 0–35 μg/mL DOX/250 nM PTX, 24 h	↓ STAT3, ERK, AKT	[46]
Gallic acid	Lung	SCLC H446 cells	2–12 µg/mL gallic acid + 3.12–50 µg/mL CDPP	↑ apoptosis, MMP disruption ↑ Bax, ↑ APAF1, ↑ p53, ↑ DIABLO, ↓ XIAP	[219]
	Breast	MCF-7/DOX cells MCF-7/DOX_500_	30–120 µM gallic acid + 5–20 µM EGCG, 24 h	↓ MMP-2/ MMP-9	[220]
	Lung	HCC827, H1650, H1975, H358, H1666 cells TKI resistant	20–100 µM gallic acid + 0.1–5 µM GF, 5 days	↓ Src-STAT3, ↑ apoptosis	[221]
Cinnamic acid	Lung	Chemoresistant H1299-derived stem-like cells	1–32 mM cinnamic acid; 4 mM cinnamic acid + 4–32 µM PTX/ 4–32 μg/mL CDPP, 24 h	↑ differentiation into CD33 negative cells; ↓chemoresistance to cisplatin and PTX	[227]
Caffeic acid/ ferulic acid	Colorectal	HCT-8 cells	Pre-treatment - 0.5–1 mg/mL BPIS (12 h) before 1000–6000 µM 5-FU, 50–400 µM OX, 25–125 µM VCR	↓ P-gp, MRP1, BCRP	[222]
Caffeic acid phenethyl ester (CAPE)	Breast	MDA-MB-231 cells	10–40 µM CAPE, 4.5 days	↓ CD44 cells, ↓ progenitor formation	[223]
	Breast	MDA-MB-231, T47D cells	Pretreatment with 1 µM CAPE (72 h) before irradiation (2–8 Gy)	↑ DNA damage	[224]
	Lung	A549 cells	10, 50 µM CAPE 10 µM DOX, 24 h	↑ chemosensitivity to DOX, ↓ claudin -2	[226]
Ellagic acid	Colorectal	SW480, Colo 320DM, HT-29 cells	5–25 µM 5-FU + 2–25 µM ellagic acid	↑ Bax/Bcl-2 ratio, ↑ caspase-3 ↓ mitochondrial potential	[217]
Sanguiin-H6	Breast	DOX resistant MCF-7	0–313 µM sanguiin-H6, 48 h	↓ ABC transporters	[218]
**Non-Flavonoid Compounds**					
Curcumin	Colorectal	OX-resistant HTOXAR3, LoVOXAR3 DLDOXAR3	5–10 μM curcumin + 10–30 μM OX, – 24 h	↓NF-κB signaling cascade, ↓ CXCL8, CXCL1, CXCL2	[230]
	Colorectal	VCR resistant HCT8/VCR	6.25–100 μM curcumin + 0.5 μg/l VCR, 48 h	↓ P-gp	[228]
	Colorectal	5-FU and OX resistant HCT-116, SW-620	100 nM CDF	↓ miR-21	[232]
	Lung	A549-CDPP resistant	20 μg/mL CDDP + 10 μM curcumin, 24 h	↓ autophagy, ↓ Nrf2 activation	[233]
	Lung	A549/DOX cells, P-gp overexpressing DOX resistant overexpressing	Nanomicelles with 1–30 μg/mL DOX + curcumin (1.6 times concentration of DOX), 72 h	↑ sensitivity to DOX, ↑ cellular uptake	[234]
	Lung	CDPP resistant A549 cells	5–20 μM curcumin + 1.5 μg/mL CDPP	↑ apoptosis, ↓ HIF-1α	[235]
	Breast	Tamoxifen resistant MCF-7/LCC2, MCF-7/LCC9	30 μM curcumin, 24 h	↓ mTOR, ↓ EZH2	[236]
	Breast	MCF-7, MDA-MB-231, SK-BR-3 cells	10 μM curcumin 6 h before 5-FU (10 μM)	↓ NF-κB signaling cascade	[231]
	Breast	DOX resistant MCF-7 cells	0–20 mM curcumin + 0–4 mΜ EGCG	↓ Bcl-2, ↓ survivin, ↑ caspase 7, 9	[238]
	Breast	MDA-MB-231, MDA-MB-468, SK-BR-3, MCF-7 cells	30 μM curcumin and/or 1 μM trans retinoic acid, 48 h	↑ sensitivity to retinoic acid ↓ FBAP5, PPARβ/δ	[237]
Gingerol	Prostate	DOC resistant PC3	100 µM 6-gingerol + 100 µM 10-gingerol	↓ MRP1, ↓GST	[51]
	Breast	cyclophosphamide, 5-5-FU, DOX resistant MCF-7	50–250 µM 6-gingerol	↓ Wnt/β-catenin, ↓ GSK3	[239]

Legend: 5-FU—5-fluorouracil, CDF—difluorinated curcumin, ↓—downregulation, ↑—upregulation, m-TOR—mammalian target of rapamycin, EZH2—enhancer of zeste homolog 2, CDPP—cisplatin, Nrf2—erythroid 2-related factor 2, DOX—doxorubicin (adriamycin), EGCG—epigallocatechingallate, Bcl-2—Bcl-lymphoma 2, Bax—Bcl-2-like protein 4, MRP1/2—multidrugresistance associated protein 1/2, GST—gluthatione-S transferase, GSK3—glycogen synthase kinase 3, AKT—protein kinase B, RES—resveratrol, P-gp—P-glycoprotein (MDR1), PTX—paclitaxel, BCRP—breast cancer resistant protein, GF—gefitinib, HER-2—human epidermal growth factor 2, HNK—honokiol, MMP—mitochondrial membrane potential, APAF1—apoptotic protease activating factor 1, DIABLO—second mitochondria-derived activator of caspases, XIAP—inhibitor of apoptosis protein 3, MMP-2/MMP-9—metalloproteinase, TKI—tyrosine kinase inhibitors (gefitinib), SChA—schizandrin A, SECO—secoisolariciresinol, ENL—enterolactone, DOC—docetaxel, CAB—carboplatin, FAS—fatty acid synthase, CSC—cancer stem cells, OX—oxalipaltin, VCR—vincristine, FBAP5—fatty acid-binding protein 5, PPARβ/δ—peroxisome proliferator-activated receptor β/δ, HIF-1α—hypoxia-inducible factor 1 alpha, NSCLC—non-small cell lung cancer, EMT—epithelial to mesenchymal transition, CREB -1—element binding protein-1, STAT3—signal transducer and activator of transcription 3, ERK—extracellular-signal regulated kinase, EGFR—epidermal growth factor receptor, CDK—cyclin-dependent kinase, IAP—inhibitors of apoptosis proteins, cFLIPL—regulator of caspase-8 activation, ATR—protein kinase, p-53—cellular tumor antigen, Chk1/2—Check point kinase 1/2, ROS—reactive oxygen species, YB-1—Y-box binding protein, CPT11—irinotecan, PI3K/AKT—phosphoinositide 3-kinase/protein kinase B, JNK—c-Jun N-terminal kinase, GRP78—glucose regulated protein, Axl, Tyro3—receptors for tyrosine kinase, TRAIL—TNF-related apoptosis-inducing ligand, NA—not applicable, C—catechin, Nf-kb—nuclear factor kappa-light-chain-enhancer of activated B cells, IGF-1R—insulin growth factor, EGCG—epigallocatechingallate, Her2/neu—receptor tyrosine-proteinkinase erB-2, XIAP—inhibitor of apoptosis protein 3, Src- proto-oncogene tyrosine-protein kinase, BPIS—bound polyphenols of inner shell from foxtail millet bran, CAPE—caffeic acid phenethyl ester, ABC—ATP-binding cassette transporter proteins.

**Table 3:** Summary of in vivo and clinical experiments.

Compound	Type of Cancer	Model System	Doses and Duration of Administration	Mechanisms of Overcoming MDR	Reference
**Flavonoid Compounds**					
Quercetin	Breast	Female Sprague–Dawley rats	1.5, 7.5, 10 mg/kg quercetin p.o. + 10 mg/kg tamoxifen p.o.	↓ P-gp, ↓ MRP2, ↓ BCPR, ↓ CYP3A4	[257]
	Breast	Xenograft BALB/c nude mouse model for MCF-7 DOX resistant cells	5 mg/kg BNDQ i.v. 20 days, every three days	↓ P-gp	[258]
Wogonin	Lung	Xenograft mouse model for A549 cells	3 mg/kg TRAIL i.p. + 100 mg/kg wogonin i.p. 3 times/week, 28 days	↑ ROS, ↑ apoptosis, ↓ cFLIP_L_, ↓ XIAP, ↓ cIAP-1, ↓ IAP-2	[41]
Fisetin	Colorectal	Xenograft nude mouse model for Lovo OX/irinotecan resistant cells	400 mg/kg/day fisetin and 800 mg/kg/day fisetin p.o., 4 weeks	↑ apoptosis, ↑ cytochrome C release, ↓ IGF1R/AKT, ↓ tumor volumes	[196]
Luteolin	Lung	Xenograft BALB/c nude mouse model for NCI-H1975 erlotinib resistant cells	10/30 mg/kg/day luteolin i.p. + 100 mg/kg/day erlotinib i.p. + 2 mg/kg/day CDPP i.p., 15 days	↓ tumor volumes, ↓ EGFR, ↓ PI3K/AKT mTOR ↑ apoptosis	[259]
Genistein	Lung	Xenograft mouse models for A549 cells	5 mg/kg CDPP i.p., day one + 800 μg/kg genistein p.o., 5 days, 5 mg/kg CDPP i.p. day one + 500 μg/kg genistein p.o., 4 days, every 7 days for 21 days	↓ tumor volumes, ↓ PI3/AKT	[262]
	Lung	Xenograft BALB/c mouse models for H1975 cells	50 mg/kg GF p.o. + 100 mg/kg genistein p.o., 5 weeks	↓ EGFR, ↓ mTOR, ↑ caspase -3	[263]
EGCG	Breast	Xenograft BALB/c mouse models for breast 4T1 cancer cells	EGCG 30 mg/kg/day i.v. + PTX 10 mg/kg i.v., every two days, 24 days	↑ apoptosis, ↓ GRP78, ↓ JNK phosphorylation	[260]
	Breast	Female Sprague–Dawley rats treated with DMBA	5 mg/kg PTX i.p. + 10 mg/kg EGCG i.p., twice/week, 4 weeks	↓ CD44 cells, ↓ VEGF, ↓ MMP-2, ↑ caspase-3	[261]
**Non-Flavonoid Compounds**					
Resveratrol	Breast	Xenograft BALB/c mouse model for MCF-7/Adr resistant cells	Liposomes with 8 mg/kg PTX + 20 mg/kg RES i.v., every two days, 14 days	↑ cellular uptake of PTX, ↓ P-gp	[264]
	Colorectal	Xenograft BALB/c nude mouse model for HCT-116 cells	100 mg/kg RES + 10 mg/kg OX i.v. every day, 14 days	↑ miR-34c	[265]
	Lung	Xenograft BALB/c mouse model (females) for SPC-A-1/CDDP cells	1 g/kg/day RES p.o., 3 g/kg/day RES p.o., 28 days	↓ survivin, ↑ apoptosis (caspase 3)	[266]
Caffeic acid phenethyl exter (CAPE)	Breast	Xenograft Ncr-*nu/nu* mouse models for MCF-7, MDA-MB-213 cells	10, 50, 250 nmol/mouse CAPE p.o., every day, 60 days	↓ NF-κB, ↓ EGFR, IFGR, ↓ MDR1	[267]
Podophyllotoxin (PPT)	Breast and prostate	Xenograft BALB/c and NOD-SCID mouse models EMT6/AR1 (breast), PC3 (prostate) cells	12 mg/kg DOC i.v., every 4 days, 8 days; 5 mg/kg CBZ i.v., every 4 days, 8 days; 180 mg/kg PPT NPs i.v. every 4 days, 8 days	↓ P-gp, ↑ cellular uptake of chemotherapeutic agents	[44]
Deoxypodophyllotoxin (DPPT)	Breast	Xenograft mouse model MCF-7 DOX resistant cells	1.25 mg/kg DPPT i.v. + 12.5 mg/kg PTX i.v. every 3 days, 10 days	efflux transport	[45]
Silybin	Breast	Xenograft mouse model (females) for MDA-MB-231 cells	1.5 mg/kg nanosystems − 75 μg/mg DOX + 120 μg/mg PTX + 90 μg/mg silybin i.v. every 4 days, 30 days	P-gp	[268]
Curcumin	Colorectal	HCT-116 cells in orthotopic mouse model	1 g/kg curcumin by gavage, daily + 60 mg/kg capecitabine by gavage, twice weekly, 4 weeks	↓ NF-κB, ↓MMP-2, ↓ CXCR4, ↓ COX-2, ↓ ICAM-1, ↓VEGF	[269]
	Colorectal	Swiss albino rats with N-Nitroso N-methyl urea–induced carcinogenesis	Pre-treatment with curcumin 50 mg/kg p.o. for one week before administration of irinotecan 30 μg/mL i.v.	↓ P-gp, ↑ sensitivity of cancer cells to irinotecan	[270]
	Colorectal	Xenograft mouse model (6–8 weeks, females) for HCT-116 cells	1.13% Meriva (equivalent to 0.2% curcuminoids) p.o. + 7.5 mg/kg OX i.v. daily, 21 days	↓ cancer stem cells, ↓ DNA damage repair	[271]
	Prostate	Xenograft BALB/c mouse model for PC3 cells	NPs with 5 mg/kg DOC + 10 mg/kg curcumin i.v. daily, 21 days	↑ intracellular accumulation of DOC	[272]
	Prostate	Xenograft *nu/nu* mouse models (males, 5–6 weeks old) for PC-3A cells	NP with 6 mg/kg DOX + 24 mg/kg curcumin i.v. twice every three days, 4 weeks	↓ MDR, MRP	[273]
	Prostate	Xenograft mouse model for PC3 cells (nude mice)	5 mg/kg curcumin p.o. daily, 4 weeks + 160 mg/kg gemcitabine i.p. every 7 days, 21 days + 3 Gy radiation days 4, 6, 10 for 21 days	↓ MDM2	[274]
	Lung	Xenograft mouse model for A549 cells	200 mg/kg/day PS + 500 mg/kg/day curcumin p.o., 36 days	↑ pharmacokinetics ↑ accumulation in cancer tissue, ↓ P-gp, ↓ MRP1/2	[275]
	Breast	Xenograft BALB/c mouse model (6–8 weeks) for MCF-7 cell lines	NPs with Tf-PEG-CUR/DOX—50 mg/kg CUR/DOX i.v. once/week, 7 weeks	↑ cellular uptake of DOX	[276]
	Prostate	CRPC patients, non-randomized open-label phase II trial (*n* = 30)	75 mg/m^2^ DOC i.v. day 1 every 21 days for 6 cycles + 8 mg dexamethasone p.o. 12 h, 3 h and 1 h before DOC administration + 5 mg prednisone p.o. twice/day starting on day 1 + 6000 mg curcumin p.o. 7 days in each cycle	↓ PSA (50% of patients), ↓ NSE (30% of patients), suggested mechanisms: ↓ NF-κB, ↓ AR, ↓ VEGFR, ↓ MDR1B	[277]
	Breast	Advanced-metastatic breast cancer patients, single institution open-label phase I trials (*n* = 13)	100 mg/m^2^ DOC i.v. day 1 of each 3 weeks cycle for 6 cycles + 450 mg curcumin p.o. 7 days consecutive for each cycle + 50 mg methylprednisolone 2 days before and after chemotherapy	↓ CEA, ↓ VEGF suggested mechanisms: ↓ P-gp	[278]

Legend—↓—downregulation, ↑—upregulation, COX-2—cicloxygenase 2, MMP-2—metalloproteinase, ICAM-1—intercellular adhesion molecule 1, CXCR4 chemokine receptor type 4, VEGF—vascular endothelial growth factor, DOC—docetaxel, P-gp—P-glycoprotein (MDR1), PS—phospho-sulindac, MRP1/2—multidrugresistance associated protein 1/2, Meriva—turmeric/phospholipid formulation, MDM2—mouse double minute 2 homolog, DOX—doxorubicin (adryamicin), Tf-PEG-CUR—transferrin-poly(ethylene glycol)-curcumin, PTX—paclitaxel, EGFR—epidermal growth factor receptor, EGR-1—early growth response protein 1, MDR—multidrug resistance, CBZ—cabazitaxel, CYP3A4—cytochrome P450 3A4, AKT—protein kinase B, XIAP—inhibitor of apoptosis protein 3, BCRP—breast cancer resistance protein, IGF-1R—insulin growth factor 1 receptor, IAP—inhibitors of apoptosis proteins, cFLIPL—regulator of caspase-8 activation, GRP78—glucose regulated protein, PI3K/AKT—phosphoinositide 3-kinase/protein kinase B, AR—androgen receptor, mTOR—mammalian target of rapamycin, NSCLC—non-small cell lung cancer, p.o.—oral administration, i.v.—intravenous administration, i.p.—intraperitoneal administration, BNDQ—quercetin and doxorubicin co-encapsulated biotin receptor-targeting nanoparticles, NPs—nanoparticles, CRPC—castration-resistant prostate cancer, CgA—chromogranin, NSE—neuron-specific enolase, DMBA—7,12-dimethylbenz[a]anthracene, OX—oxaliplatin, CDPP—cisplatin, GF—gefitinib, RES—resveratrol, PPTNPs—podophyllotoxin nanoparticles, CEA—carcioembryonic antigen, TRAIL—TNF-related apoptosis-inducing ligand, ROS—reactive oxygen species, JNK—c-Jun N-terminal kinase, RES—resveratrol, CAPE—caffeic acid phenethyl ester, Nf-kb- nuclear factor kappa-light-chain-enhancer of activated B cells, DPPT—deoxypodophyllotoxin, PSA—prostate serum antigen.

## Abbreviations

↑	upregulation
↓	downregulation
5-FU	5-fluorouracil
ABC	ATP-binding cassette transporter proteins
ABCB1, ABCG1	isoforms of ATP-binding cassette transporter proteins
ARE	antioxidant response element
ABT-263	small molecule that inhibits Bcl-2; 4-(4-{[2-(4-Chlorophenyl)-5,5-dimethyl-1-cyclohexen-1-yl]methyl}c-1-piperazinyl)-N-[(4-{[(2R)-4-(4-morpholinyl)-1-(phenylsulfanyl)-2-butanyl]amino}-3-[(trifluoromethyl)sulfonyl]phenyl)sulfonyl]benzamide
ABT-737	small molecule that inhibits Bcl-2; 4-{4-[(4′-Chloro-2-biphenylyl)methyl]-1-piperazinyl}-N-[(4-{[(2R)-4-(dimethylamino)-1-(phenylsulfanyl)-2-butanyl]amino}-3-nitrophenyl)sulfonyl]benzamide
AKT	protein kinase B
ALDH	aldehyde dehydrogenase
ALP	autophagy lysosomes systems
AMPK	AMP-activated protein kinase
APAF1	apoptotic protease activating factor 1
APR-246	drug that binds to p53 (restoring p53 function) and depletes glutathione; PRIMA-1, 2-hydroxymethyl-2-methoxymethyl-aza-bicyclo[2.2.2]octan-3-one
AR	androgen receptor
ATR	serine/threonine protein kinase
Axl, Tyro3	receptors for tyrosine kinase
BASE	base excision repair
Bax	Bcl-2-associated X protein/Bcl-2-like protein 4
Bcl-2	B cell lymphoma 2 protein
Bcl-XL	B cell lymphoma extra-large protein
BCRA1, 2	breast cancer susceptible genes
BCRP	breast cancer resistant protein
BER	base excision repair
BH	Bcl-2 homology domain
BIM	Bcl-2 like protein 11
BNDQ	quercetin and doxorubicin co-encapsulated biotin receptor-targeting nanoparticles
BPIS	bound polyphenols of inner shell from foxtail millet bran
BRAF	serine/threonine-protein kinase B-Raf
C	catechin
CAB	carboplatin
CAPE	caffeic acid phenethyl ester
CAR	constitutive androstane receptor
caspase-3, 8, 9	cysteine aspartic proteases-3, 8, 9
CBZ	cabazitaxel
CD44, 24, 133	cluster of differentiation 44, 24, 133
CDF	difluorinated curcumin
CDK 2,4,6	cyclin-dependent kinases 2,4,6
CDPP	cisplatin
CEA	carcioembryonic antigen
cFLIP	regulator of caspase-8 activation; cellular FLICE (FADD-like IL-1β-converting enzyme)-inhibitory protein
CgA	chromogranin
Chk1/2	Check point kinase 1/2
cIAP-1,2	cellular inhibitor of apoptosis protein 1,2
COMT	catechol-O-methyl transferase
COX-2	ciclo-oxygenase 2
CPT11	irinotecan
CREB-1	element binding protein-1
CRPC	castration-resistant prostate cancer
CSC	cancer stem cells
CXCR4	CXC chemokine receptor type 4
CYP1A1, CYP1B1, CYP19A1, CYP17A1	isoforms of cytochrome 450
CYP3A4	cytochrome P450 3A4
DDR	DNA damage response
DIABLO	direct IAP-binding protein with Low pI
DMBA	7,12-dimethylbenz[a] anthracene
DNA	deoxyribonucleic acid
DOC	docetaxel
DOX	doxorubicin (adriamycin)
DPPT	deoxypodophyllotoxin
DR4/5	pro-apoptotic death receptors
EC	epicatechin
EGC	epigallocatechin
EGCG	epigallocatechingallate
EGF	epidermal growth factor
EGFR	epithelial growth factor receptor
EGFR(T790M)	epithelial growth factor receptor with a mutation that replace threonine by methionine at position 790
EGR-1	early growth response protein 1
EMT	epithelial-mesenchymal transition
ENL	enterolactone
ER	estrogen receptors
ERα/ERβ	estrogen receptor alpha/estrogen receptor beta
ERK 1,2	extracellular-signal regulated kinase
ETS2	proto-oncogene 2, transcription factor (v-ets, Avian Erythroblastosis Virus E26 Oncogene Homolog 2)
EZH2	enhancer of zeste homolog 2 (histone methyltransferase)
FASN	fatty acid synthase
FBAP5	fatty acid-binding protein 5
FGF	fibroblast growth factor
FOLFOX	5-fluorouracil, oxaliplatin, folinic acid
GF	gefitinib
GRP78	glucose regulated protein
GSH	reduced glutathione
GSK3	glycogen synthase kinase 3
GST	glutathione-S transferase
GPX	glutathione peroxidase
GTE	green tea extract
HER-2, 3	human epidermal growth factor 2, 3
Her2/neu	receptor tyrosine-proteinkinase erbB-2
HGFR/MET	hepatocyte growth factor receptor
HIF-1α	hypoxia-inducible factor 1 alpha
hMLH1	mismatch repair gene of human mutL homolog 1
HNK	honokiol
HO-1	hemeoxygenase 1
HR	homologous recombination
HRas	transforming protein p21
IAP	inhibitors of apoptosis proteins
ICAM-1	intercellular adhesion molecule 1
IGF-1R	insulin growth factor receptor
IL-6, 8, 17, 18	interleukin-6, 8, 17, 18
i.p.	intraperitoneal administration
i.v	intravenous administration
JNK	c-Jun N-terminal kinase
Keap 1	kelch-like ECH-associated protein 1
KRAS	gene identified in Kirsten rat sarcoma
MAPK	mitogen activated protein kinase
MDM2	mouse double minute 2 homolog
MDR	multidrug resistance
Meriva	turmeric/phospholipid formulation
MET	tyrosine-proteinkinase
MLH1,2	human mutL homolog 1,2
MMP	mitochondrial membrane potential
MMP-2,9	metalloproteinases 2,9
MMR	mismatch repair
MRP1/2	multidrug resistance associated protein 1/2
mRNA	messenger RNA
miRNA	microRNA
miR-16, 17, 21, 200c, 17-5p, 892c	microRNA-16, 17, 21, 200c, 17-5p, 892c
MSH 1/2	DNA mismatch repair protein 1/2
mTOR	mammalian target of rapamycin
NAD(P)H	reduced nicotinamide adenine dinucleotide phosphate
NER	nucleotide excision repair
NF-κB	nuclear factor kappa-light-chain-enhancer of activated B cells
NGF	nerve growth factor
NHEJ	non-homologous end-joining
NOX	NADPH oxidases
NPs	nanoparticles
Nrf2	erythroid 2-related factor 2
NSCLC	non-small cell lung cancer
NSE	neurospecific enolase
OX	oxaliplatin
p53	tumor suppressor protein
P-gp (MDR1)	P-glycoprotein (multidrug resistance protein 1)
PI3K/AKT	phosphoinositide 3-kinase/protein kinase B
PKC	proteinkinase C
p.o.	oral administration
PPARβ/δ	peroxisome proliferator-activated receptor β/δ
PPT	podophyllotoxin
PS	phospho-sulindac
PSA	prostate serum antigen
PTEN	phosphatase and tensin homolog
PTX	paclitaxel
PXR	pregnane X receptor
RARs	retinoic acid receptors
RES	resveratrol
ROS	reactive oxygen species
SCC	squamous cell carcinoma
SChA	schizandrin A
SCLC	small cell lung cancer
SECO	secoisolariciresinol
Smac	second mitochondria-derived activator of caspase
SOD	superoxide-dismutase
Src	proto-oncogene tyrosine-protein kinase
SRT501	small molecule, a form of resveratrol designed to target sirtuin 1 protein
STAT3	signal transducer and activator of transcription 3
T-box 3	T-box transcription factor 3
Tf-PEG-CUR	transferrin-poly(ethylene glycol)-curcumin
TGF-β	transforming growth factor
TKI	tyrosine kinase inhibitors
TME	tumor microenvironment
TP53	gene coding tumor suppressor protein p53
TRAIL	TNF-related apoptosis-inducing ligand
UGT	uridine diphospho-glucuronosyltransferase
VCR	vincristine
VEGF	vascular endothelial growth factor
VEGFR2	vascular endothelial growth factor receptor 2
Wnt/β-catenin	wingless-type MMTV integration site family member (MMTV, mouse mammary tumor virus)/beta-catenin signaling pathway
xCT	glutamate cysteine antiporter
XIAP	X-linked inhibitor of apoptosis protein
YB-1	Y-box binding protein-1
